# Association Between Adherence to the Japanese Meal-based Dietary Guideline and All-cause and Cause-specific Mortalities: A Japan Public Health Center-based Prospective Study

**DOI:** 10.2188/jea.JE20240495

**Published:** 2026-01-05

**Authors:** Mariko Takano, Junko Ishihara, Ayaka Kotemori, Kumiko Kito, Fumi Hayashi, Yukari Takemi, Hiroyasu Iso, Kazumasa Yamagishi, Taiki Yamaji, Motoki Iwasaki, Manami Inoue, Shoichiro Tsugane, Norie Sawada

**Affiliations:** 1 Department of Nutrition Science, Graduate School of Kagawa Nutrition University, Saitama, Japan; 2 Department of Food and Life Science, School of Life and Environmental Science, Azabu University, Kanagawa, Japan; 3 Research Fellow of Japan Society for the Promotion of Science, Tokyo, Japan; 4 Division of Cohort Research, National Cancer Center Institute for Cancer Control, Tokyo, Japan; 5 Institute for Global Health Policy Research, Bureau of International Cooperation, Japan Institute for Health Security, Tokyo, Japan; 6 Department of Public Health Medicine, Institute of Medicine, and Health Services Research and Development Center, University of Tsukuba, Ibaraki, Japan; 7 Department of Public Health, Graduate School of Medicine, Juntendo University, Tokyo, Japan; 8 Division of Epidemiology, National Cancer Center Institute for Cancer Control, Tokyo, Japan; 9 International University of Health and Welfare Graduate School of Public Health, Tokyo, Japan

**Keywords:** meal-based dietary guidelines, mortality, Japan, prospective study

## Abstract

**Background:**

The Ministry of Health, Labour, and Welfare in Japan has published a meal-based dietary guideline (Healthy Meal); however, its relationship with health outcomes remains unclear. This observational study examined the association between adherence to Healthy Meal and all-cause and cause-specific mortalities.

**Methods:**

We analyzed data from the Japan Public Health Center-based Prospective Study (JPHC Study) with a mean follow-up of 19.0 years, including 40,222 men and 47,350 women aged 45–75 years with no history of cancer, stroke, ischemic heart disease, chronic liver disease, or kidney disease. Adherence to Healthy Meal was scored using dietary intake from a validated food frequency questionnaire. Cox proportional hazard models were used to estimate the hazard ratios (HRs) and 95% confidence intervals (CIs) for all-cause and cause-specific mortalities across score quartiles.

**Results:**

A higher Healthy Meal adherence score was significantly associated with a lower risk of all-cause mortality. The multivariable-adjusted HRs for the highest versus the lowest adherence group were 0.86 (95% CI, 0.82–0.91, *P* < 0.001 for trend) in men and 0.92 (95% CI, 0.87–0.98, *P* = 0.005 for trend) in women. Significant associations with a lower risk of cerebrovascular disease and respiratory disease mortalities were observed in both sexes. In contrast, significant associations were observed for cancer, cardiovascular disease, and heart disease mortalities in men only.

**Conclusion:**

Higher adherence to the Japanese meal-based dietary guideline was associated with a lower risk of all-cause, cerebrovascular disease, and respiratory disease mortalities in Japanese men and women, and cancer, cardiovascular disease, and heart disease mortalities in men only.

## INTRODUCTION

Many countries have established dietary guidelines to promote adequate nutrient intake, improve health, and prevent noncommunicable diseases. Recently, measures to evaluate adherence to dietary guidelines have been developed to explore their relationship with health outcomes. In the United States, the Healthy Eating Index measures diet quality based on compliance with the Dietary Guidelines for Americans^1^^–^^3^ and is associated with a lower risk of mortality and cardiovascular diseases (CVDs).^4^^,^^5^ In Japan, the Ministry of Health, Labour, and Welfare and the Ministry of Agriculture, Forestry, and Fisheries developed the Japanese Food Guide Spinning Top (JFGST) in 2005.^6^ Adherence to the JFGST is inversely related to mortality.^7^^,^^8^

These dietary guidelines, which have been reported to improve health outcomes, recommend adequate daily intake. However, some countries, such as the United States, use MyPlate,^9^ which provides meal-based guidelines to help individuals easily understand the appropriate amount to eat for each meal. In Japan, the Ministry of Health, Labour, and Welfare published a meal-based dietary guideline, Healthy Meal for Japanese individuals, in 2015.^10^ This guideline recommends dietary intake primarily based on dishes that improve individual health and prevent lifestyle-related diseases. Furthermore, it provides a standard for the amount of meals served to promote a healthy food environment. The recommended intake per meal was determined such that the nutrient intake meets 30% of the daily recommended amounts based on the Dietary Reference Intakes for Japanese individuals, closely aligning with the current diet. However, evidence of the health benefits of adhering to Healthy Meal has not been provided.

In this study, we aimed to evaluate the association between adherence to Healthy Meal and all-cause and cause-specific mortalities in a prospective cohort study of Japanese men and women.

## METHODS

### Study design

The Japan Public Health Center-based Prospective Study (JPHC Study) commenced in 1990 for cohort I and in 1993 for cohort II.^11^ Participants in cohort I were residents aged 40–59 years from five Japanese public health center areas (Iwate, Akita, Nagano, Okinawa-Chubu, and Tokyo), whereas cohort II consisted of residents aged 40–69 years from six other public health center areas (Ibaraki, Niigata, Kochi, Nagasaki, Okinawa-Miyako, and Osaka). A self-administered questionnaire was utilized to gather data on the participants’ medical histories and health-related lifestyles, including smoking, drinking, and dietary habits, at baseline and at the 5- and 10-year follow-ups (second and third surveys, respectively). The baseline characteristics of the participants were primarily derived from the second survey, whereas medical histories were obtained from both the baseline and second surveys. In cases where the responses differed between the two surveys, participants who answered “yes” to having a medical history in either survey were considered to have the condition. For dietary intake, we used data from the second survey, which included more detailed information collected through a semi-quantitative food frequency questionnaire (FFQ). The protocol of the present study was approved by the institutional review boards of the National Cancer Center, Japan (ethical approval number: 2001-021), and Azabu University (approval number: 2527). Participants were informed of the objectives of the study, and that completion of the survey questionnaire was regarded as providing consent to participate.

### Study population

Of 140,420 participants at baseline, 103,514 responded to the second survey. We excluded 1,088 participants with missing FFQ data, 5,118 with extreme energy intake (top and bottom 2.5% of sex-specific distribution: 985–4,185 kcal for men, 838–3,686 kcal for women), and 9,736 with a history of cancer (*n* = 2,906), stroke (*n* = 1,140), ischemic heart disease (*n* = 2,623), chronic liver disease (*n* = 2,181), or chronic kidney disease (*n* = 1,701). Ultimately, the data of 87,572 participants (40,222 men and 47,350 women) were analyzed.

### Dietary assessment

The dietary assessment was performed using the FFQ from the second survey, which included 147 food and beverage items and nine frequency categories.^12^ Participants were asked to report the consumption frequency and portion size for most foods. The validity and reproducibility of the FFQ have been demonstrated in previous studies.^13^^–^^16^ Briefly, 247 participants in cohort I and 392 in cohort II were voluntarily enrolled in the validation study. They were asked to complete the FFQ twice within a 1-year interval, along with 7 consecutive days of weighted dietary records (DRs) in 4 seasons, totaling 28 days. The first and second FFQ results were compared for reproducibility, and the second FFQ was compared with the DRs for validity.

### Score calculation based on Healthy Meal adherence

We created a score to evaluate adherence to Healthy Meal,^10^ based on six components: grain dishes (amount of carbohydrates from grains), fish and meat dishes (amount of protein from seafood, meat, eggs, and soybeans), vegetable dishes (amount of vegetables, potatoes, mushrooms, and seaweed intake), milk and dairy products, fruits, and salt equivalents. Table 1 presents the guideline standards and calculation methods. The recommended intake of four components, excluding milk and dairy products and fruits, was defined per single meal in two categories: <650 kcal and ≥650 kcal. The energy of a single meal was calculated based on one-third of the energy derived from grain, meat and fish, and vegetable dishes. To calculate the scores, we compared one-third of the total daily intake of grain, meat and fish, and vegetable dishes as well as the salt equivalent to the standard. We used the total daily intake of milk and dairy products and fruits to calculate the scores, as the guideline assumes they are consumed at some point during the day. This recommendation is based on the Japanese dietary pattern, where milk and dairy products and fruits are often consumed as snacks. Notably, snacking comprises approximately 10% of the total energy intake.^17^ Most energy comes from grains, meat and fish, and vegetable dishes in the three main meals.^17^ Each component was scored on a 0–1 point scale; 1 point was allotted if criteria were met, and points were deducted according to the percentage above or below the reference range. For the salt equivalent, points were deducted only if its consumption exceeded the upper limit. For components whose intake was below the lower limit of the standard range, the score was calculated using the following formula: Score = 1 × (consumed amount)/(lower limit of the standard range). For intakes above the upper limit, the score was calculated as: Score = 1 − [(consumed amount) − (upper limit of standard range)]/upper limit of standard range. The formulas used to calculate the scores were based on the score for JFGST adherence in a previous study.^8^ The scores for each of the six components were then summed to obtain a total score (range: 0–6 points).

**Table 1.** Components of Healthy Meal and scoring system

Component	Score^a^	Standard of food/nutrient intake		Crude standard^b^		Corrected standard^c^	
	
			Men	<650 kcal	≥650 kcal	<526 kcal	≥526 kcal
			Women			<530 kcal	≥530 kcal
Grain dishes	0–1	Carbohydrate from grains (g/day/3^d^)	Men	40–70	70–95	47.4–65.1	65.1–79.8
			Women			48.7–63.3	63.3–75.4

Fish and meat dishes	0–1	Protein from seafood, meat, eggs, and soybeans (g/day/3^d^)	Men	10–17	17–28	11.3–14.0	14.0–18.2
			Women			10.9–14.6	14.6–20.5

Vegetable dishes	0–1	Vegetable, potato, mushroom, seaweed, and bean intake (g/day/3^d^)	Men	120–200	120–200	101.9–142.3	101.9–142.3
			Women			119.0–168.2	119.0–168.2

Milk and dairy products	0–1	Milk and dairy product intake (g/day)	Men	100–200	100–200	196.7–317.8	196.7–317.8
			Women			181.2–311.8	181.2–311.8

Fruits	0–1	Fruit intake (g/day)	Men	100–200	100–200	192.5–301.1	192.5–301.1
			Women			214.7–315.1	214.7–315.1

Salt equivalent	0–1	Salt equivalent intake (g/day/3^d^)	Men	<3.0	<3.5	<3.5	<3.9
			Women			<3.5	<3.9

Total	0–6						

^a^If an individual consumed less than standard range, scores were calculated as follows: 1 * (consumed amount of food or nutrient)/(lower limit of standard range).

If an individual consumed more than standard range, scores were calculated as follows: 1 − [(consumed amount of food or nutrient) − (upper limit of standard range)]/(upper limit of standard range).

These formulas were based on a previous study (Kurotani K, et al 2016).

^b^The values were recommended by Japanese meal-based dietary guideline (Healthy Meal).

^c^The values were corrected standard estimated by regression equations between dietary intakes obtained from food frequency questionnaires and dietary records.

^d^To calculate the scores, we compared one-third of the total daily intake of grain, meat, and fish dishes; vegetable dishes; and salt equivalent to the standard.

To calculate scores based on dietary intake from the FFQs, we assessed the validity and reproducibility using FFQs and DRs from the validation study. Of the participants in the validation study, 245 men and 259 women with no missing DRs were analyzed. We corrected for the cutoff value for energy intake and the six components using regression equations between dietary intakes obtained from FFQs and DRs, as FFQ dietary data were not objective values. The derived regression equations are presented in eTable 1. The scores were calculated using the corrected reference value of a category defined by the corrected cutoff value for energy intake. Consistency between each score calculated from FFQs using the corrected cutoff values and DRs using the original values was examined using Spearman correlation and Cohen *κ* analyses. The Spearman rank correlation coefficients were 0.40 for men and 0.32 for women. The Cohen *κ* coefficients, based on the score quartiles, were 0.82 for men and 0.81 for women. Reproducibility was examined with Spearman correlation between the first and second FFQs, yielding coefficients of 0.50 for men and 0.59 for women.

### Follow-up and outcome

The residency and vital status of the participants were followed using the residential registry from baseline to December 31, 2018, for all public health centers except Osaka and Tokyo, where the study periods ended on December 31, 2012, and December 31, 2009, respectively. The causes of death were determined using death certificates and defined according to the International Classification of Diseases, Tenth version (ICD-10).^18^ The primary cause of death was applied to each participant. The major endpoints were all-cause mortality, cancer (ICD-10 codes, C00–C97), CVD (I00–I99), heart disease (I20–I52), cerebrovascular disease (I60–I69), and respiratory disease (J10–J18 and J40–J47).

### Statistical analysis

We determined the follow-up period in person-years for each participant, beginning from the date they responded to the second questionnaire and ending at the earliest occurrence of death, emigration from Japan, or December 31, 2018. The Healthy Meal adherence scores were adjusted for energy intake using the residual method (average intake: 2,161 kcal for men and 1,857 kcal for women). The participants were categorized into quartiles according to the sex-specific distributions of the energy-adjusted score. Descriptive analyses were performed on the score distribution (energy-adjusted, crude, and component scores), the dietary intake used for score calculation, and basic characteristics.

Hazard ratios (HRs) and 95% confidence intervals (CIs) for mortality according to the score quartile were estimated using an adjusted Cox proportional hazards model. A linear trend was also observed. The first (lowest) quartile was used as the reference. HRs and 95% CIs were also estimated for a 1-point increase in the energy-adjusted score. Model 1 adjusted for age (years, continuous) and study area (11 areas); model 2 added adjustments for body mass index (BMI) (<18.5, 18.5–24.9, 25.0–29.9, ≥30.0 kg/m^2^), smoking status (lifetime non-smoker, former smoker, or current smoker with a consumption of <20 or ≥20 cigarettes/day), alcohol consumption (0, 1–149, 150–299, ≥300 g of ethanol equivalent/week), total physical activity (sex-specific quartile of metabolic equivalent task-h/day), history of or medication usage for diabetes mellitus, hypertension, and dyslipidemia (yes or no), occupation (agriculture, forestry, fishery, office work, self-employed, specialty work, housewife, unemployed, or other; yes or no), coffee consumption (almost never, <1, or ≥2 cups/day; 1 cup = 120 mL), and green tea consumption (almost never, <1, 1, 2–3, ≥4 cups/day). Model 3 was further adjusted for the total energy intake (kcal/day, continuous). Missing data were handled using indicator variables. To address the concern of adjusting only for baseline age, we performed a sensitivity analysis using age as the time scale for proportional-hazards regression. However, the results did not differ significantly from those obtained using the original model. Therefore, we retained the original model for consistency across our cohort studies.

We also analyzed data stratified by alcohol consumption (<300 g/week/≥300 g/week in only men), smoking status (not current/current), and occupation (agriculture, fishery, and forestry/others) as sensitivity analyses. To assess the statistical interactions, we added an interaction term created by multiplying the score (continuous) and stratifying the variables (dichotomous) to the model.

All statistical analyses were performed using SAS version 9.4 (SAS Institute Inc., Cary, NC, USA). Statistical significance was defined as a two-sided *P*-value of <0.05.

## RESULTS

The distributions of the energy-adjusted and crude scores (total and by component), and food and nutrient intake according to the quartile groups are shown in Table 2, eTable 2, and eTable 3. Median energy-adjusted scores were 3.2 (interquartile range [IQR], 2.9–3.4) in men and 3.4 (IQR, 3.1–3.6) in women for the lowest group, and 5.0 (IQR, 4.8–5.2) in men and 5.1 (IQR, 5.0–5.3) in women for the highest group. Large score differences between the lowest and highest groups were observed for vegetable dishes, milk and dairy products, and fruits. Basic characteristics are shown in Table 3. Participants with higher scores were more likely to have a normal BMI (18.5–24.9 kg/m^2^), be non-smokers, and consume less alcohol.

**Table 2.** Total score and its components according to energy-adjusted score for adherence to Healthy Meal

		Men (*n* = 40,222)				Women (*n* = 47,350)			
	
		Quartile of energy-adjusted score for adherence to Healthy Meal				Quartile of energy-adjusted score for adherence to Healthy Meal			
	
		Q1 (low) (*n* = 10,055)	Q2 (*n* = 10,056)	Q3 (*n* = 10,056)	Q4 (high) (*n* = 10,055)	Q1 (low) (*n* = 11,837)	Q2 (*n* = 11,838)	Q3 (*n* = 11,838)	Q4 (high) (*n* = 11,839)
(Energy-adjusted) Total score		3.2 (2.9, 3.4)	3.9 (3.7, 4.0)	4.4 (4.3, 4.5)	5.0 (4.8, 5.2)	3.4 (3.1, 3.6)	4.1 (4.0, 4.2)	4.6 (4.5, 4.7)	5.1 (5.0, 5.3)

(Crude value)									
Total score		3.2 (2.8, 3.4)	3.9 (3.7, 4.0)	4.4 (4.2, 4.5)	5.0 (4.8, 5.2)	3.4 (3.0, 3.7)	4.1 (3.9, 4.3)	4.6 (4.5, 4.8)	5.2 (5.0, 5.4)

Grain dishes		1.0 (0.7, 1.0)	1.0 (0.8, 1.0)	1.0 (0.9, 1.0)	1.0 (1.0, 1.0)	1.0 (0.9, 1.0)	1.0 (1.0, 1.0)	1.0 (1.0, 1.0)	1.0 (1.0, 1.0)
Fish and meat dishes		0.6 (0.4, 0.9)	0.9 (0.6, 1.0)	1.0 (0.8, 1.0)	1.0 (0.9, 1.0)	0.6 (0.4, 0.9)	0.9 (0.6, 1.0)	0.9 (0.8, 1.0)	1.0 (0.9, 1.0)
Vegetable dishes		0.4 (0.2, 0.7)	0.5 (0.3, 0.8)	0.6 (0.4, 0.9)	0.7 (0.5, 0.9)	0.5 (0.3, 0.8)	0.7 (0.4, 0.9)	0.7 (0.5, 1.0)	0.8 (0.6, 1.0)
Milk and dairy products		0.1 (0.0, 0.4)	0.4 (0.1, 0.8)	0.7 (0.4, 1.0)	1.0 (0.8, 1.0)	0.1 (0.0, 0.5)	0.5 (0.1, 0.9)	0.8 (0.4, 1.0)	0.9 (0.8, 1.0)
Fruits		0.2 (0.1, 0.5)	0.5 (0.3, 0.9)	0.7 (0.4, 1.0)	1.0 (0.8, 1.0)	0.3 (0.0, 0.6)	0.6 (0.3, 0.9)	0.8 (0.5, 1.0)	1.0 (0.8, 1.0)
Salt equivalent		0.8 (0.1, 1.0)	0.8 (0.3, 1.0)	0.8 (0.4, 1.0)	0.8 (0.6, 1.0)	0.8 (0.0, 1.0)	0.8 (0.3, 1.0)	0.8 (0.5, 1.0)	0.9 (0.6, 1.0)

Total energy	(kcal/day)	1,461 (1,976, 2,735)	1,666 (2,086, 2,623)	1,759 (2,115, 2,527)	1,812 (2,093, 2,414)	1,195 (1,678, 2,460)	1,406 (1,779, 2,258)	1,494 (1,781, 2,141)	1,592 (1,798, 2,044)
Energy from grain, fish and meat, and vegetable dishes	(kcal/day/3)	307 (415, 571)	342 (425, 538)	358 (428, 517)	367 (422, 488)	274 (353, 491)	306 (373, 462)	323 (378, 449)	342 (390, 445)
Amount of carbohydrate from grain dishes	(g/day/3)	59.1 (48.9, 81.4)	59.0 (50.6, 75.8)	59.4 (51.2, 72.5)	58.1 (51.2, 69.2)	51.0 (39.8, 60.6)	52.2 (42.9, 60.2)	52.8 (44.2, 60.0)	53.2 (45.7, 59.8)
Amount of protein from fish and meat dishes	(g/day/3)	9.4 (5.7, 19.3)	11.5 (7.8, 17.2)	12.2 (9.0, 16.5)	12.3 (9.8, 15.4)	9.0 (5.4, 18.2)	10.9 (7.4, 16.0)	11.3 (8.3, 15.2)	11.8 (9.5, 14.8)
Amount of vegetable intake	(g/day/3)	47.3 (26.2, 90.9)	63.9 (40.8, 101.9)	72.3 (49.5, 105.0)	86.8 (63.6, 115.5)	68.8 (37.5, 142.3)	80.4 (52.3, 125.2)	85.3 (60.5, 119.2)	95.7 (71.9, 122.9)
Amount of milk and dairy product intake	(g/day)	21.4 (2.5, 200.0)	78.8 (23.3, 236.9)	142.0 (57.9, 226.9)	178.0 (110.0, 215.5)	72.9 (13.9, 364.3)	184.2 (50.9, 311.1)	200.0 (100.0, 269.4)	193.2 (121.9, 228.2)
Amount of fruit intake	(g/day)	36.8 (17.6, 135.8)	70.9 (36.0, 171.3)	96.6 (53.9, 170.6)	124.9 (86.9, 176.9)	118.6 (34.5, 396.2)	151.6 (68.5, 309.0)	148.7 (86.7, 242.4)	151.4 (108.8, 201.6)
Amount of salt equivalent intake	(g/day/3)	3.5 (2.1, 5.7)	3.7 (2.6, 5.2)	3.7 (2.8, 4.8)	3.5 (2.9, 4.3)	3.6 (2.0, 5.9)	3.6 (2.5, 5.1)	3.5 (2.7, 4.5)	3.4 (2.8, 4.1)

Value: median (25, 75 percentile).

**Table 3.** Basic characteristics of participants^a^ according to energy-adjusted score for adherence to Healthy Meal

		Men (*n* = 40,222)					Women (*n* = 47,350)				
	
		Quartile of energy-adjusted score for adherence to Healthy Meal				*P* value^b^	Quartile of energy-adjusted score for adherence to Healthy Meal				*P* value^b^
	
		Q1 (low) (*n* = 10,055)	Q2 (*n* = 10,056)	Q3 (*n* = 10,056)	Q4 (high) (*n* = 10,055)		Q1 (low) (*n* = 11,837)	Q2 (*n* = 11,838)	Q3 (*n* = 11,838)	Q4 (high) (*n* = 11,837)	
Range of energy-adjusted score		0.2–3.6	3.6–4.1	4.1–4.7	4.7–6.2	—	0.1–3.8	3.8–4.4	4.4–4.8	4.8–6.2	—

Age (years)		55 (49, 62)	55 (49, 62)	55 (49, 62)	55 (50, 62)	<0.001	57 (51, 63)	56 (50, 62)	56 (50, 62)	55 (49, 61)	<0.001

Body Mass Index (kg/m^2^)	<18.5	317 (3.2)	288 (2.9)	271 (2.7)	278 (2.8)	<0.001	489 (4.1)	442 (3.7)	438 (3.7)	455 (3.8)	<0.001
	18.5–24.9 (normal)	6,677 (66.4)	6,740 (67.0)	6,802 (67.6)	6,851 (68.1)		7,519 (63.5)	7,795 (65.8)	8,053 (68.0)	8,052 (68.0)	
	25.0–29.9	2,523 (25.1)	2,602 (25.9)	2,572 (25.6)	2,583 (25.7)		2,922 (24.7)	2,907 (24.6)	2,761 (23.3)	2,809 (23.7)	
	30.0≤	227 (2.3)	224 (2.2)	211 (2.1)	163 (1.6)		386 (3.3)	378 (3.2)	352 (3.0)	317 (2.7)	

Total physical activity (metabolic equivalents h/day)		31.9 (26.1, 36.1)	31.9 (26.7, 36.1)	31.9 (27.1, 36.1)	31.9 (27.1, 36.1)	<0.001	31.9 (26.1, 34.3)	31.9 (27.1, 34.3)	31.9 (27.1, 34.3)	31.9 (27.1, 34.3)	<0.001

Smoking status	Non-smoker	3,028 (30.1)	3,195 (31.8)	3,463 (34.4)	3,814 (37.9)	<0.001	9,944 (84.0)	10,334 (87.3)	10,513 (88.8)	10,569 (89.3)	<0.001
	Former smoker	1,417 (14.1)	1,676 (16.7)	1,764 (17.5)	1,857 (18.5)		130 (1.1)	120 (1.0)	98 (0.8)	126 (1.1)	
	Current (<20 cigarettes/day)	3,216 (32.0)	3,092 (30.7)	2,837 (28.2)	2,725 (27.1)		707 (6.0)	612 (5.2)	528 (4.5)	506 (4.3)	
	Current (≥20 cigarettes/day)	1,764 (17.5)	1,611 (16.0)	1,529 (15.2)	1,241 (12.3)		73 (0.6)	67 (0.6)	46 (0.4)	45 (0.4)	

Alcohol consumption (Ethanol equivalent)	0 (g/week)	2,670 (26.6)	2,500 (24.9)	2,397 (23.8)	2,283 (22.7)	<0.001	9,389 (79.3)	9,285 (78.4)	9,202 (77.7)	8,979 (75.9)	<0.001
	1–149 (g/week)	2,188 (21.8)	2,446 (24.3)	2,581 (25.7)	2,686 (26.7)		1,558 (13.2)	1,854 (15.7)	2,007 (17.0)	2,268 (19.2)	
	150–299 (g/week)	1,797 (17.9)	1,851 (18.4)	1,910 (19.0)	2,056 (20.4)		264 (2.2)	244 (2.1)	227 (1.9)	225 (1.9)	
	300≤ (g/week)	3,249 (32.3)	3,161 (31.4)	3,066 (30.5)	2,949 (29.3)		156 (1.3)	131 (1.1)	123 (1.0)	121 (1.0)	

History or Medication (Yes or No for each item)	Hypertension	2,102 (20.9)	2,100 (20.9)	2,168 (21.6)	2,475 (24.6)	<0.001	2,669 (22.5)	2,624 (22.2)	2,607 (22.0)	2,553 (21.6)	0.336
	Diabetes	734 (7.3)	813 (8.1)	878 (8.7)	1,008 (10.0)	<0.001	522 (4.4)	475 (4.0)	488 (4.1)	522 (4.4)	0.308
	Dyslipidemia	341 (3.4)	381 (3.8)	411 (4.1)	420 (4.2)	0.017	817 (6.9)	952 (8.0)	864 (7.3)	859 (7.3)	0.008

Occupation (Yes or No for each item)	Agriculture	2,372 (23.6)	2,293 (22.8)	2,287 (22.7)	2,185 (21.7)	0.019	2,657 (22.4)	2,454 (20.7)	2,383 (20.1)	2,331 (19.7)	<0.001
	Forestry	124 (1.2)	101 (1.0)	84 (0.8)	53 (0.5)	<0.001	29 (0.2)	21 (0.2)	20 (0.2)	17 (0.1)	0.305
	Fishery	378 (3.8)	333 (3.3)	260 (2.6)	267 (2.7)	<0.001	57 (0.5)	53 (0.4)	28 (0.2)	36 (0.3)	0.004
	Salaried	3,210 (31.9)	3,827 (38.1)	3,860 (38.4)	3,982 (39.6)	<0.001	2,325 (19.6)	2,552 (21.6)	2,695 (22.8)	2,824 (23.9)	<0.001
	Self-employed	1,783 (17.7)	1,796 (17.9)	1,807 (18.0)	1,789 (17.8)	0.976	1,261 (10.7)	1,343 (11.3)	1,332 (11.3)	1,376 (11.6)	0.113
	Specialty work	827 (8.2)	918 (9.1)	922 (9.2)	861 (8.6)	0.048	449 (3.8)	555 (4.7)	551 (4.7)	617 (5.2)	<0.001
	Housework	19 (0.2)	14 (0.1)	22 (0.2)	17 (0.2)	0.595	4,646 (39.2)	4,832 (40.8)	5,160 (43.6)	5,170 (43.7)	<0.001
	Unemployed	893 (8.9)	835 (8.3)	908 (9.0)	1,076 (10.7)	<0.001	1,100 (9.3)	1,030 (8.7)	897 (7.6)	891 (7.5)	<0.001
	Other	858 (8.5)	750 (7.5)	730 (7.3)	638 (6.3)	<0.001	984 (8.3)	968 (8.2)	964 (8.1)	960 (8.1)	0.946

Green tea consumption	Almost none	2,336 (23.2)	2,131 (21.2)	1,994 (19.8)	1,751 (17.4)	<0.001	2,641 (22.3)	2,317 (19.6)	2,163 (18.3)	1,929 (16.3)	<0.001
	<1 cup/day	1,919 (19.1)	2,106 (20.9)	2,094 (20.8)	2,115 (21.0)		1,947 (16.4)	2,170 (18.3)	2,199 (18.6)	2,213 (18.7)	
	1 cup/day	920 (9.1)	1,052 (10.5)	1,069 (10.6)	1,132 (11.3)		992 (8.4)	1,101 (9.3)	1,079 (9.1)	1,208 (10.2)	
	2–3 cups/day	1,923 (19.1)	2,069 (20.6)	2,165 (21.5)	2,357 (23.4)		2,123 (17.9)	2,414 (20.4)	2,584 (21.8)	2,684 (22.7)	
	≧4 cups/day	2,218 (22.1)	2,373 (23.6)	2,494 (24.8)	2,495 (24.8)		3,316 (28.0)	3,406 (28.8)	3,482 (29.4)	3,550 (30.0)	

Coffee consumption	Almost none	3,123 (31.1)	2,994 (29.8)	2,727 (27.1)	2,729 (27.1)	<0.001	3,478 (29.4)	3,294 (27.8)	2,991 (25.3)	2,734 (23.1)	<0.001
	<1 cup/day	3,099 (30.8)	3,387 (33.7)	3,515 (35.0)	3,466 (34.5)		3,464 (29.3)	3,883 (32.8)	4,070 (34.4)	4,199 (35.5)	
	1 cup/day	1,298 (12.9)	1,513 (15.0)	1,560 (15.5)	1,707 (17.0)		1,996 (16.9)	2,092 (17.7)	2,224 (18.8)	2,378 (20.1)	
	≧2 cups/day	1,540 (15.3)	1,653 (16.4)	1,894 (18.8)	1,875 (18.6)		1,804 (15.2)	2,047 (17.3)	2,143 (18.1)	2,210 (18.7)	

Energy intake (kcal)		1,976 (1,461, 2,735)	2,086 (1,666, 2,623)	2,115 (1,759, 2,527)	2,093 (1,812, 2,414)	<0.001	1,678 (1,195, 2,460)	1,779 (1,406, 2,258)	1,780 (1,494, 2,141)	1,798 (1,592, 2,044)	<0.001

Value: median (25, 75 percentile) for age, total physical activity, and energy intake; *n* (%) for BMI, smoking status, alcohol consumption, history or medication, occupation, green tea consumption, and coffee consumption.

^a^Participants with missing data excluded (men, women): 893, 1,275 for BMI; 1,993, 2,932 for smoking status; 432, 1,317 for alcohol consumption, 1,832, 1,509 for green tea consumption, 2,343, 2,142 for coffee consumption.

^b^Chi-square test was used for categorical variables; Kruskal-Wallis test was used for continuous variables.

During the 1,661,348 person-years of follow-up (mean 19.0 years), we identified 20,671 all-cause deaths (12,370 men and 8,301 women). Table 4 shows the HRs and 95% CIs for all-cause, cancer, CVD, heart disease, cerebrovascular disease, and respiratory disease mortalities by score quartile. The score was inversely associated with the risk of all-cause mortality in both sexes. The multivariable-adjusted HRs for the highest group were 0.86 (95% CI, 0.82–0.91, *P* < 0.001 for trend) in men and 0.92 (95% CI, 0.87–0.98, *P* = 0.005 for trend) in women. HRs of the 1-point increment were 0.92 (95% CI, 0.90–0.95) in men and 0.96 (95% CI, 0.94–0.99) in women. Significant associations with lower risk of cancer, CVD, and heart disease mortalities were observed in men only; the multivariable-adjusted HRs for the highest group were 0.91 (95% CI, 0.84–0.99, *P* = 0.004 for trend), 0.80 (95% CI, 0.72–0.89, *P* < 0.001 for trend), and 0.84 (95% CI, 0.73–0.96, *P* = 0.003 for trend), respectively. The risk of cerebrovascular disease mortality decreased with a higher score in men; the multivariable-adjusted HR for the highest group was 0.74 (95% CI, 0.62–0.87, *P* < 0.001 for trend). In women, significant reductions in the HRs for the highest group and 1-point increment were observed; however, there was no linear association by quartile (*P* = 0.068 for trend). The score was inversely associated with respiratory disease mortality in both sexes (*P* = 0.021 and 0.010 for trend, respectively), with significant reductions in HRs for the highest group and a 1-point increment observed only in women.

**Table 4.** Multivariable adjusted hazard ratios (HR) and 95% confidence intervals of mortality according to energy-adjusted score for adherence to Healthy Meal

	Men (*n* = 40,222)						Women (*n* = 47,350)					
	
	Quartile of energy-adjusted score for adherence to Healthy Meal					1 point increment	Quartile of energy-adjusted score for adherence to Healthy Meal					1 point increment
	
	Q1 (low) (*n* = 10,055)	Q2 (*n* = 10,056)	Q3 (*n* = 10,056)	Q4 (high) (*n* = 10,055)	*P* for trend		Q1 (low) (*n* = 11,837)	Q2 (*n* = 11,838)	Q3 (*n* = 11,838)	Q4 (high) (*n* = 11,839)	*P* for trend	
Person years	182,104	183,553	185,790	184,677	—	—	231,083	231,822	231,695	230,624	—	—
All-cause mortality												
Number of deaths	3,437	3,185	2,903	2,845	—	—	2,405	2,141	1,960	1,795	—	—
Model 1^a^	1.00 (ref)	0.93 (0.89–0.98)	0.82 (0.78–0.86)	0.79 (0.76–0.83)	<0.001	0.88 (0.86–0.90)	1.00 (ref)	0.97 (0.91–1.02)	0.91 (0.85–0.96)	0.87 (0.82–0.93)	<0.001	0.94 (0.91–0.96)
Model 2^b^	1.00 (ref)	0.98 (0.93–1.03)	0.88 (0.83–0.92)	0.86 (0.82–0.91)	<0.001	0.92 (0.90–0.95)	1.00 (ref)	0.99 (0.94–1.05)	0.95 (0.90–1.01)	0.92 (0.87–0.98)	0.006	0.97 (0.94–0.99)
Model 3^c^	1.00 (ref)	0.98 (0.93–1.03)	0.88 (0.83–0.92)	0.86 (0.82–0.91)	<0.001	0.92 (0.90–0.95)	1.00 (ref)	0.99 (0.94–1.05)	0.95 (0.90–1.01)	0.92 (0.87–0.98)	0.005	0.96 (0.94–0.99)
Cancer mortality												
Number of deaths	1,304	1,219	1,118	1,141	—	—	729	743	661	639	—	—
Model 1^a^	1.00 (ref)	0.94 (0.87–1.01)	0.83 (0.77–0.90)	0.84 (0.78–0.91)	<0.001	0.91 (0.87–0.94)	1.00 (ref)	1.07 (0.97–1.19)	0.97 (0.88–1.08)	0.98 (0.88–1.09)	0.335	0.99 (0.94–1.04)
Model 2^b^	1.00 (ref)	0.97 (0.90–1.05)	0.88 (0.81–0.96)	0.91 (0.84–0.99)	0.004	0.95 (0.91–0.98)	1.00 (ref)	1.09 (0.98–1.20)	1.00 (0.90–1.11)	1.00 (0.90–1.11)	0.627	1.00 (0.95–1.06)
Model 3^c^	1.00 (ref)	0.97 (0.90–1.05)	0.88 (0.81–0.96)	0.91 (0.84–0.99)	0.004	0.95 (0.91–0.98)	1.00 (ref)	1.09 (0.98–1.20)	1.00 (0.90–1.11)	1.00 (0.90–1.11)	0.626	1.00 (0.95–1.06)
Cardiovascular disease mortality												
Number of deaths	904	788	719	690	—	—	703	590	551	518	—	—
Model 1^a^	1.00 (ref)	0.88 (0.80–0.97)	0.77 (0.70–0.85)	0.73 (0.66–0.81)	<0.001	0.86 (0.82–0.90)	1.00 (ref)	0.92 (0.83–1.03)	0.89 (0.80–0.99)	0.89 (0.79–0.99)	0.023	0.94 (0.89–0.99)
Model 2^b^	1.00 (ref)	0.93 (0.85–1.03)	0.83 (0.75–0.92)	0.80 (0.72–0.89)	<0.001	0.90 (0.86–0.94)	1.00 (ref)	0.96 (0.86–1.07)	0.95 (0.85–1.06)	0.95 (0.85–1.07)	0.378	0.98 (0.92–1.03)
Model 3^c^	1.00 (ref)	0.93 (0.85–1.03)	0.83 (0.75–0.92)	0.80 (0.72–0.89)	<0.001	0.90 (0.86–0.95)	1.00 (ref)	0.96 (0.86–1.07)	0.95 (0.84–1.06)	0.95 (0.85–1.07)	0.368	0.98 (0.92–1.03)
Heart disease mortality												
Number of deaths	469	416	371	378	—	—	359	320	269	282	—	—
Model 1^a^	1.00 (ref)	0.90 (0.79–1.02)	0.77 (0.67–0.88)	0.77 (0.68–0.89)	<0.001	0.88 (0.83–0.94)	1.00 (ref)	0.99 (0.85–1.16)	0.86 (0.73–1.01)	0.95 (0.81–1.11)	0.231	0.97 (0.90–1.04)
Model 2^b^	1.00 (ref)	0.94 (0.83–1.08)	0.82 (0.72–0.94)	0.84 (0.73–0.97)	0.003	0.93 (0.87–0.99)	1.00 (ref)	1.05 (0.90–1.22)	0.93 (0.79–1.09)	1.05 (0.89–1.23)	0.980	1.02 (0.95–1.10)
Model 3^c^	1.00 (ref)	0.94 (0.83–1.08)	0.82 (0.72–0.94)	0.84 (0.73–0.96)	0.003	0.93 (0.87–0.99)	1.00 (ref)	1.05 (0.90–1.22)	0.93 (0.79–1.09)	1.05 (0.89–1.23)	0.963	1.02 (0.95–1.10)
Cerebrovascular disease mortality												
Number of deaths	357	302	280	246	—	—	280	224	232	179	—	—
Model 1^a^	1.00 (ref)	0.85 (0.73–0.996)	0.76 (0.65–0.89)	0.66 (0.56–0.78)	<0.001	0.81 (0.75–0.87)	1.00 (ref)	0.86 (0.73–1.03)	0.93 (0.78–1.10)	0.76 (0.63–0.92)	0.014	0.88 (0.81–0.96)
Model 2^b^	1.00 (ref)	0.91 (0.78–1.07)	0.83 (0.71–0.97)	0.73 (0.62–0.87)	<0.001	0.86 (0.80–0.92)	1.00 (ref)	0.89 (0.75–1.06)	0.97 (0.82–1.16)	0.81 (0.67–0.97)	0.075	0.90 (0.83–0.99)
Model 3^c^	1.00 (ref)	0.91 (0.78–1.07)	0.83 (0.71–0.98)	0.74 (0.62–0.87)	<0.001	0.86 (0.80–0.93)	1.00 (ref)	0.89 (0.74–1.06)	0.97 (0.81–1.16)	0.80 (0.66–0.97)	0.068	0.90 (0.83–0.99)
Respiratory disease mortality												
Number of deaths	291	316	263	244	—	—	188	163	136	99	—	—
Model 1^a^	1.00 (ref)	1.09 (0.93–1.28)	0.86 (0.72–1.01)	0.79 (0.66–0.93)	<0.001	0.89 (0.82–0.96)	1.00 (ref)	0.98 (0.79–1.21)	0.83 (0.66–1.03)	0.65 (0.51–0.83)	<0.001	0.83 (0.75–0.92)
Model 2^b^	1.00 (ref)	1.16 (0.99–1.37)	0.93 (0.79–1.11)	0.87 (0.73–1.03)	0.021	0.94 (0.87–1.01)	1.00 (ref)	1.03 (0.84–1.28)	0.91 (0.73–1.14)	0.73 (0.57–0.93)	0.010	0.88 (0.79–0.98)
Model 3^c^	1.00 (ref)	1.16 (0.99–1.37)	0.93 (0.79–1.11)	0.87 (0.73–1.03)	0.021	0.93 (0.86–1.01)	1.00 (ref)	1.03 (0.84–1.28)	0.91 (0.73–1.14)	0.73 (0.57–0.93)	0.010	0.88 (0.79–0.98)

Adjusted hazard ratios and 95% confidence intervals. Analysis using Cox proportional hazards model.

^a^Model1 was adjusted for age and study area (11 areas).

^b^Model2 was adjusted as for model1 plus body mass index, smoking status, alcohol drinking, total physical activity, history or medication of hypertension/diabetes/dyslipidemia, occupation, green tea consumption and coffee consumption.

^c^Model3 was adjusted as for model2 plus energy intake.

The results of the stratified analyses by alcohol consumption, smoking status, and occupation are shown in eTable 4, eTable 5, eTable 6, eTable 7, eTable 8 and eTable 9. We found significant interactions in alcohol consumption for mortality due to cerebrovascular disease in men (*P* = 0.012 for interaction) and in smoking status for mortality due to CVD and heart disease in women (*P* = 0.014 and 0.010 for interaction, respectively). No significant interactions were observed in smoking status in men or occupation in men and women.

## DISCUSSION

In this large prospective study, we examined the relationship between adherence to Healthy Meal in the Japanese population and all-cause and cause-specific mortalities. The main differences between the high and low Healthy Meal Adherence score groups were observed in milk and dairy product intake and fruit intake, while differences in grain and salt intake were relatively small. However, this study primarily evaluated the effect of the combination with six components of Healthy Meal guidelines as a whole. Our results showed that higher adherence to the guidelines was significantly associated with lower risks of all-cause, cerebrovascular disease, and respiratory disease mortalities in both sexes. In contrast, the risks of cancer, CVD, and heart disease mortalities were significantly reduced only in men.

In the present study, the risk of cerebrovascular disease mortality decreased with increased adherence to Healthy Meal in both sexes. This decrease was partly explained by the higher fruit and vegetable consumption among those with higher adherence to Healthy Meal. Meta-analyses have shown inverse associations between fruit and vegetable intake and the risk of stroke in Japan^19^ and other countries.^20^ Sodium consumption, a major contributor to stroke,^21^^,^^22^ can also explain this association. Although the median salt equivalent scores were similar across quartiles, the higher adherence group had higher 25 percentile scores (ie, lower consumption), potentially contributing to reduced cerebrovascular disease mortality. Given that adherence to the JFGST, which includes components similar to Healthy Meal such as vegetables and fruits, was not significantly associated with lower cerebrovascular disease mortality in men,^8^ the inclusion of reference salt intake levels in Healthy Meal may help explain this difference in outcomes. Higher fat intake with a higher consumption of fish and meat dishes and milk and dairy products can also explain this association. In Japan, where fat consumption is lower than that in Western countries,^23^ higher intakes of saturated and animal fats were reported to be inversely associated with the risk of stroke, especially cerebral hemorrhage,^24^ and cerebral infarction mortality,^25^ respectively. The results of this study support this unique inverse association in the Japanese population.

A risk reduction in respiratory disease mortality was observed only in Q4 among women, whereas no risk reduction was observed across the groups in men, with only a linear association observed. The most common cause of death due to respiratory disease was pneumonia (75% of men and 85% of women). Outbreaks and the severity of pneumonia have been reported to be caused by poor nutritional status^26^ and frailty.^27^ Thus, higher adherence to Healthy Meal might have been associated with a reduction in poor nutritional status, leading to a lower risk of pneumonia.

An inverse association between adherence to Healthy Meal and cancer mortality was found only in men, likely because of their higher intake of fruits, vegetables, and milk and dairy products. Fruit and vegetable consumption was reported to reduce the risk of cancer mortality.^20^ Our cohort study has also found a marginally significant association between a higher intake of fruits and vegetables and cancer mortality.^28^ Additionally, milk and dairy consumption was non-linearly associated with reduced cancer mortality in men, with those consuming the appropriate amount (a median of about 150 g/day) having the lowest risk.^29^ In this study, a 1-point increment of milk and dairy products significantly decreased cancer mortality by 11% in men (data not shown). The significant association observed only in men may be partly explained by sex differences in the frequencies of some types of cancers, as supported by previous findings.^8^ Our cohort data have suggested that fruit and vegetable consumption protects against gastric,^30^ esophageal,^31^ and extrahepatic bile duct cancers.^32^ Increased fruit intake also reduced the risk of pancreatic cancer,^33^ and dietary fiber, rich in fruits and vegetables, reduced the risk of lung cancer in men.^34^ Furthermore, a higher consumption of dairy products and calcium is protective against colorectal cancer.^35^^,^^36^ In this study, these cancers contributed more to all-cancer mortalities in men (67%) than in women (55%), possibly explaining the sex-specific reduction in cancer mortality.

We also observed a significant inverse association between adherence to Healthy Meal and CVD and heart disease mortalities in men only. In the Japanese population, higher consumption of fruits and vegetables and higher potassium intake have been identified as diet-related factors contributing to a lower risk of CVDs.^37^ In this study, the higher adherence group consumed more fruits and vegetables rich in potassium than those consumed in the lower adherence group, which might have caused an inverse association between the score and CVD mortality in men. No significant associations were observed among women, particularly those who were not currently smoking, which may be attributed to women consuming more fruits and vegetables than men. The 75^th^ percentile fruit and vegetable intake value for the lowest group was higher than that in the other higher groups in women.

The strengths of our study are its large-scale prospective cohort design, high rate of participant response, long follow-up, and the application of an FFQ with established validity. However, this study has several limitations. First, although Healthy Meal provides a meal-based standard of dietary intake, we could not assess the dietary intake per meal using the FFQ. We did not account for the uneven distribution of meal sizes across the three meals.^17^ This may have led to inaccurate assessments of mortality risk reduction. Second, changes in dietary habits were not considered because we used dietary intake data from only one time point. However, a previous study^8^ within the same cohort showed moderate correlations in dietary intake between follow-up surveys conducted 5 years apart, suggesting reasonable stability in dietary patterns over time. Third, we did not use reference values based on individual energy requirements because of a lack of objective basal metabolic rates or physical activity data. Moreover, the correlation coefficients between the scores derived from the FFQ and the DR were low to modest. Nevertheless, a significant correlation was observed, supporting the validity of these scores. Given the low validity of the FFQ, any association observed with health outcomes may be attenuated,^38^ potentially resulting in an underestimation of the risk reduction.

In conclusion, we found that Japanese men and women with a higher adherence to Healthy Meal, a meal-based dietary guideline for Japanese individuals, had a lower risk of mortality from all causes, cerebrovascular disease, and respiratory disease. Although further studies are needed, a well-balanced diet composed of an appropriate intake of grains, fish and meat, vegetables, milk and dairy products, fruits, and salt equivalents can be one of the healthy diet indicators that decrease the risk of mortality.

## SUPPLEMENTARY MATERIAL

eTable 1. The regression equation to correct the cutoff point of the reference value

eTable 2A. Total score and its components according to energy-adjusted score for adherence to Healthy Meal in men

eTable 2B. Total score and its components according to energy-adjusted score for adherence to Healthy Meal in women

eTable 3A. Food and nutrient intake accordiing to adherence to Healthy Meal in men

eTable 3B. Food and nutrient intake accordiing to adherence to Healthy Meal in women

eTable 4. Multivariable adjusted hazard ratios and 95% confidence intervals of mortality according to energy-adjusted score for adherence to Healthy Meal stratified by alcohol consumption in men

eTable 5. Multivariable adjusted hazard ratios and 95% confidence intervals of mortality according to energy-adjusted score for adherence to Healthy Meal stratified by alcohol consumption in women

eTable 6. Multivariable adjusted hazard ratios and 95% confidence intervals of mortality according to energy-adjusted score for adherence to Healthy Meal stratified by smoking status in men

eTable 7. Multivariable adjusted hazard ratios and 95% confidence intervals of mortality according to energy-adjusted score for adherence to Healthy Meal stratified by smoking status in women

eTable 8. Multivariable adjusted hazard ratios and 95% confidence intervals of mortality according to energy-adjusted score for adherence to Healthy Meal stratified by occupation in men

eTable 9. Multivariable adjusted hazard ratios and 95% confidence intervals of mortality according to energy-adjusted score for adherence to Healthy Meal stratified by occupation in women
