# Supplementary material for: Association Between Adherence to the Japanese Meal-based Dietary Guideline and All-cause and Cause-specific Mortalities: A Japan Public Health Center-based Prospective Study
Source: J Epidemiol. 2026 Jan 5;36(1):35–43. doi: 10.2188/jea.JE20240495 (PMC12698322; doi:10.2188/jea.JE20240495)
Supplement: Supplementary file 1 [file je-36-035-s001.pdf]

eTable 1. The regression equation to correct the cutoff point of the reference value

Component	The regression equation (men)	The regression equation (women)
Energy	y = 0.4958x + 203.05	y = 0.5174x + 193.99
Grain dishes	y = 0.59x + 23.78	y = 0.4849x + 29.317
Fish and meat dishes	y = 0.3819x + 7.4712	y = 0.5317x + 5.5805
Vegetable dishes	y = 0.5057x + 41.198	y = 0.6154x + 45.135
Milk and dairy products	y = 1.2109x + 75.597	y = 1.3053x + 50.701
Fruits	y = 1.0863x + 83.875	y = 1.0035x + 114.38
Salt equivalent	y = 0.7091x + 1.3729	y = 0.7191x + 1.3544

DR, dietary record; FFQ, food frequency questionnaire; x, consumed amount assessed using DR; y, consumed amount assessed using FFQ.

eTable 2A. Total score and its components according to energy-adjusted score for adherence to Healthy Meal in men

		Men (n=40,222)																			
		Quartile of energy-adjusted score for adherence to Healthy Meal																			
		Q1 (low) (n=10,055)					Q2 (n=10,056)					Q3 (n=10,056)					Q4 (high) (n=10,055)				
	Percentile	P5	P25	P50	P75	P95	P5	P25	P50	P75	P95	P5	P25	P50	P75	P95	P5	P25	P50	P75	P95
(Energy-adjusted) Total score		2.3	2.9	3.2	3.4	3.5	3.6	3.7	3.9	4.0	4.1	4.2	4.3	4.4	4.5	4.6	4.7	4.8	5.0	5.2	5.6
(Crude value)																					
Total score		2.2	2.8	3.2	3.4	3.7	3.5	3.7	3.9	4.0	4.2	4.0	4.2	4.4	4.5	4.7	4.6	4.8	5.0	5.2	5.6
Grain dishes		0.4	0.7	1.0	1.0	1.0	0.5	0.8	1.0	1.0	1.0	0.6	0.9	1.0	1.0	1.0	0.7	1.0	1.0	1.0	1.0
Fish and meat dishes		0.0	0.4	0.6	0.9	1.0	0.3	0.6	0.9	1.0	1.0	0.5	0.8	1.0	1.0	1.0	0.6	0.9	1.0	1.0	1.0
Vegetable dishes		0.1	0.2	0.4	0.7	1.0	0.2	0.3	0.5	0.8	1.0	0.2	0.4	0.6	0.9	1.0	0.3	0.5	0.7	0.9	1.0
Milk and dairy products		0.0	0.0	0.1	0.4	1.0	0.0	0.1	0.4	0.8	1.0	0.0	0.4	0.7	1.0	1.0	0.4	0.8	1.0	1.0	1.0
Fruits		0.0	0.1	0.2	0.5	1.0	0.0	0.3	0.5	0.9	1.0	0.1	0.4	0.7	1.0	1.0	0.4	0.8	1.0	1.0	1.0
Salt equivalent		0.0	0.1	0.8	1.0	1.0	0.0	0.3	0.8	1.0	1.0	0.0	0.4	0.8	1.0	1.0	0.1	0.6	0.8	1.0	1.0
Total energy	(kcal/day)	1,099	1,461	1,976	2,735	3,703	1,248	1,666	2,086	2,623	3,414	1,372	1,759	2,115	2,527	3,178	1,493	1,812	2,093	2,414	2,966
Energy from grain, fish and meat, and vegetable dishes	(kcal/day/3)	214	307	415	571	782	256	342	425	538	705	277	358	428	517	654	298	367	422	488	600
Amount of carbohydrate from grain dishes	(g/day/3)	25.1	48.9	59.1	81.4	110.3	33.2	50.6	59.0	75.8	102.5	35.6	51.2	59.4	72.5	98.5	38.1	51.2	58.1	69.2	89.7
Amount of protein from fish and meat dishes	(g/day/3)	2.7	5.7	9.4	19.3	33.8	4.8	7.8	11.5	17.2	27.3	5.8	9.0	12.2	16.5	24.1	6.9	9.8	12.3	15.4	20.5
Amount of vegetable intake	(g/day/3)	9.1	26.2	47.3	90.9	242.8	20.5	40.8	63.9	101.9	190.9	27.9	49.5	72.3	105.0	174.9	39.2	63.6	86.8	115.5	169.9
Amount of milk and dairy product intake	(g/day)	0.0	2.5	21.4	200.0	750.0	0.0	23.3	78.8	236.9	648.9	10.0	57.9	142.0	226.9	516.3	51.1	110.0	178.0	215.5	294.6
Amount of fruit intake	(g/day)	1.5	17.6	36.8	135.8	544.5	13.4	36.0	70.9	171.3	428.2	23.3	53.9	96.6	170.6	330.5	46.4	86.9	124.9	176.9	261.9
Amount of salt equivalent intake	(g/day/3)	1.1	2.1	3.5	5.7	9.0	1.6	2.6	3.7	5.2	7.6	1.8	2.8	3.7	4.8	7.0	2.1	2.9	3.5	4.3	5.8

Values: 5, 25, 50, 75, 95 percentile

eTable2B. Total score and its components according to energy-adjusted score for adherence to Healthy Meal in women

		Women (n=47,350)																			
		Quartile of energy-adjusted score for adherence to Healthy Meal																			
		Q1 (low) (n=11,837)					Q2 (n=11,838)					Q3 (n=11,838)					Q4 (high) (n=11,839)				
	Percentile	P5	P25	P50	P75	P95	P5	P25	P50	P75	P95	P5	P25	P50	P75	P95	P5	P25	P50	P75	P95
(Energy-adjusted) Total score		2.5	3.1	3.4	3.6	3.8	3.8	4.0	4.1	4.2	4.3	4.4	4.5	4.6	4.7	4.8	4.9	5.0	5.1	5.3	5.7
(Crude value)																					
Total score		2.4	3.0	3.4	3.7	3.9	3.6	3.9	4.1	4.3	4.5	4.2	4.5	4.6	4.8	4.9	4.8	5.0	5.2	5.4	5.7
Grain dishes		0.5	0.9	1.0	1.0	1.0	0.6	1.0	1.0	1.0	1.0	0.7	1.0	1.0	1.0	1.0	0.8	1.0	1.0	1.0	1.0
Fish and meat dishes		0.1	0.4	0.6	0.9	1.0	0.4	0.6	0.9	1.0	1.0	0.5	0.8	0.9	1.0	1.0	0.7	0.9	1.0	1.0	1.0
Vegetable dishes		0.1	0.3	0.5	0.8	1.0	0.2	0.4	0.7	0.9	1.0	0.3	0.5	0.7	1.0	1.0	0.4	0.6	0.8	1.0	1.0
Milk and dairy products		0.0	0.0	0.1	0.5	1.0	0.0	0.1	0.5	0.9	1.0	0.0	0.4	0.8	1.0	1.0	0.4	0.8	0.9	1.0	1.0
Fruits		0.0	0.0	0.3	0.6	1.0	0.0	0.3	0.6	0.9	1.0	0.0	0.5	0.8	1.0	1.0	0.5	0.8	1.0	1.0	1.0
Salt equivalent		0.0	0.0	0.8	1.0	1.0	0.0	0.3	0.8	1.0	1.0	0.0	0.5	0.8	1.0	1.0	0.2	0.6	0.9	1.0	1.0
Total energy	(kcal/day)	922	1,195	1,678	2,460	3,285	1,103	1,406	1,779	2,258	2,935	1,213	1,494	1,781	2,141	2,704	1,353	1,592	1,798	2,044	2,508
Energy from grain, fish and meat, and vegetable dishes	(kcal/day/3)	200	274	353	491	684	229	306	373	462	620	253	323	378	449	578	285	342	390	445	541
Amount of carbohydrate from grain dishes	(g/day/3)	21.0	39.8	51.0	60.6	88.3	28.0	42.9	52.2	60.2	82.5	31.9	44.2	52.8	60.0	78.2	35.9	45.7	53.2	59.8	74.0
Amount of protein from fish and meat dishes	(g/day/3)	2.7	5.4	9.0	18.2	31.5	4.5	7.4	10.9	16.0	25.3	5.3	8.3	11.3	15.2	22.2	7.0	9.5	11.8	14.8	19.7
Amount of vegetable intake	(g/day/3)	15.7	37.5	68.8	142.3	295.9	29.1	52.3	80.4	125.2	212.0	35.8	60.5	85.3	119.2	186.1	47.1	71.9	95.7	122.9	172.3
Amount of milk and dairy product intake	(g/day)	0.0	13.9	72.9	364.3	810.0	5.0	50.9	184.2	311.1	765.3	26.5	100.0	200.0	269.4	600.0	65.0	121.9	193.2	228.2	308.2
Amount of fruit intake	(g/day)	8.0	34.5	118.6	396.2	722.3	26.3	68.5	151.6	309.0	579.6	39.8	86.7	148.7	242.4	433.4	67.3	108.8	151.4	201.6	281.0
Amount of salt equivalent intake	(g/day/3)	1.1	2.0	3.6	5.9	8.9	1.6	2.5	3.6	5.1	7.3	1.8	2.7	3.5	4.5	6.3	2.0	2.8	3.4	4.1	5.3

Values: 5, 25, 50, 75, 95 percentile

eTable 3A. Food and nutrient intake accordiing to adherence to Healthy Meal in men

		Q1					Q2					Q3					Q4				
		P5	P25	P50	P75	P95	P5	P25	P50	P75	P95	P5	P25	P50	P75	P95	P5	P25	P50	P75	P95
Grains	(g)	290.0	489.1	613.6	738.9	962.4	334.1	495.8	594.5	698.1	881.1	355.2	498.7	587.3	682.0	849.9	375.1	497.8	572.1	652.8	791.0
Potatoes	(g)	20.9	32.5	38.9	48.8	84.8	23.4	33.7	41.1	52.5	84.3	25.3	34.8	42.6	54.6	85.0	27.4	37.0	46.1	59.8	89.3
Sugar and sweetners	(g)	0.0[a]	0.1	0.7	2.2	8.5	0.0[a]	0.1	0.7	2.6	9.4	0.0[a]	0.1	0.6	2.8	9.0	0.0[a]	0.1	0.6	2.7	8.3
Legumes	(g)	9.0	48.1	71.6	102.2	205.5	14.1	49.4	73.9	106.3	193.8	18.8	50.2	74.9	106.9	189.0	22.2	52.4	77.2	112.4	205.4
Nuts and seeds	(g)	0.0[a]	0.2	1.2	2.1	7.7	0.0[a]	0.3	1.2	2.2	8.1	0.0[a]	0.5	1.2	2.3	9.3	0.0[a]	0.6	1.3	2.5	9.8
Vegetables	(g)	11.8	97.0	145.7	217.3	514.2	38.8	113.4	166.5	237.7	421.3	57.1	125.6	178.8	247.1	398.4	82.0	152.6	208.2	277.7	401.0
Fruits	(g)	0.0[a]	57.4	113.4	207.9	576.0	0.0[a]	76.0	136.8	227.7	484.0	16.0	93.5	155.2	233.1	412.5	48.4	125.7	180.4	243.4	361.9
Mushrooms	(g)	0.0[a]	2.7	5.0	8.6	24.9	0.0[a]	3.0	5.7	10.4	24.7	0.0[a]	3.3	6.4	11.4	25.9	0.2	3.9	7.8	13.6	28.0
Seaweed	(g)	0.0[a]	3.6	6.3	10.9	25.5	0.1	4.2	7.2	12.5	24.5	0.9	4.5	7.7	13.0	24.4	1.5	5.4	8.9	14.5	26.0
Fish and shellfish	(g)	8.9	55.1	81.1	119.5	244.3	15.1	58.5	84.0	117.9	201.4	22.9	61.4	86.7	117.9	181.7	27.4	63.0	87.4	114.9	164.8
Meat	(g)	0.0[a]	34.8	57.1	84.6	162.5	6.0	38.4	59.8	86.5	151.5	11.1	39.9	60.4	85.8	145.9	14.6	40.8	59.7	82.4	130.8
Eggs	(g)	0.0[a]	13.3	23.1	38.6	69.2	0.0[a]	14.4	25.7	41.4	65.2	1.4	15.4	27.6	43.1	62.1	3.9	17.2	29.9	45.5	59.5
Dairy products	(g)	0.0[a]	16.2	76.9	144.1	626.0	0.0[a]	34.7	98.5	230.3	589.6	0.0[a]	58.3	136.1	233.0	482.1	20.3	101.8	167.6	227.8	296.7
Fat and oil	(g)	2.6	7.3	10.0	13.7	23.0	3.8	8.0	10.8	14.2	21.0	4.8	8.5	11.1	14.4	20.7	5.7	9.3	11.9	14.9	20.9
Confectionaries	(g)	0.0[a]	3.1	8.7	15.6	45.4	0.0[a]	3.8	10.1	18.5	49.3	0.0[a]	4.8	11.0	19.6	50.0	0.0[a]	5.5	11.9	20.9	50.4
Alcohol beverages	(g)	0.0[a]	76.3	221.7	499.3	1106.7	0.0[a]	78.3	209.8	475.3	1032.7	0.0[a]	80.9	205.7	464.8	1012.5	0.0[a]	85.0	213.4	449.0	986.7
Non-alcohol beverages	(g)	100.6	386.2	653.3	1,056.8	1,842.0	149.2	427.9	688.6	1,060.6	1,815.9	181.0	447.5	700.8	1,069.5	1,806.2	207.9	462.7	707.9	1,062.9	1,767.0
Seasonings	(g)	1.0	4.8	7.0	9.8	18.5	1.7	5.2	7.5	10.7	18.4	2.3	5.5	7.8	10.9	18.2	2.7	5.9	8.2	11.1	17.9
Protein	(g)	47.7	63.0	72.1	82.7	108.6	50.8	65.2	73.4	82.0	98.7	52.2	66.4	74.2	81.9	94.7	53.3	66.7	74.4	81.1	90.9
Total fat	(g)	24.1	41.8	52.8	64.9	90.8	28.7	45.0	55.4	66.1	86.8	31.1	46.8	56.5	66.4	84.9	34.4	48.5	57.6	66.2	81.7
Carbohydrate	(g)	175.9	243.1	283.1	319.5	384.7	187.8	247.6	281.5	314.3	369.6	197.9	250.7	280.5	310.4	357.8	208.2	254.6	281.1	306.4	346.3
Sodium	(mg)	2,500	3,924	4,903	6,123	8,662	2,535	3,939	4,850	5,889	7,708	2,671	3,934	4,777	5,675	7,309	2,633	3,847	4,608	5,354	6,677
Pottasium	(mg)	1,384	2,053	2,461	3,027	4,478	1,580	2,211	2,611	3,083	3,990	1,732	2,308	2,680	3,108	3,800	1,893	2,458	2,824	3,182	3,781
Calcium	(mg)	151	309	407	548	1043	195	346	449	595	980	237	377	478	595	857	285	423	514	599	733
Magnecium	(mg)	193	263	305	360	498	207	274	314	362	461	218	279	319	363	448	228	289	328	370	448
Iron	(mg)	5.3	7.5	8.8	10.4	14.3	5.6	7.7	9.0	10.4	13.1	5.9	7.8	9.0	10.4	12.8	6.1	8.0	9.2	10.5	12.7
Retinol	(μg)	0[a]	233	421	692	1606	0[a]	250	454	747	1561	3	254	456	743	1456	38	257	459	738	1,343
α-carotene	(μg)	0[a]	190	362	629	1545	0[a]	213	408	741	1572	6	239	443	792	1578	64	302	552	951	1,773
β-carotene	(μg)	42	1,359	2,129	3,276	7,634	390	1,556	2,438	3,613	6,799	659	1,715	2,580	3,765	6,432	991	2,105	3,057	4,305	7,079
Vitamin D	(μg)	1.3	6.6	9.6	14.3	30.9	2.1	6.9	10.0	14.1	25.6	2.4	7.2	10.2	14.2	23.4	2.7	7.4	10.4	14.2	21.4
Vitamin C	(mg)	17	61	90	139	294	30	71	103	147	248	42	79	110	149	227	57	96	125	158	218
Saturated fat	(g)	5.6	11.4	15.4	19.8	31.1	7.1	12.7	16.5	20.8	29.8	8.1	13.4	16.9	20.9	28.7	9.3	14.1	17.2	20.4	26.4
Dietary fiber	(g)	4.1	7.9	10.3	13.6	23.8	5.0	8.7	11.2	14.1	20.5	5.8	9.2	11.6	14.2	19.4	6.9	10.3	12.5	14.9	19.1

Values: 5, 25, 50, 75, 95 percentile

All intake values were energy-adjusted using residual method.

[a] Negative values were replaced with 0.

eTable 3B. Food and nutrient intake accordiing to adherence to Healthy Meal in women

		Q1					Q2					Q3					Q4				
		P5	P25	P50	P75	P95	P5	P25	P50	P75	P95	P5	P25	P50	P75	P95	P5	P25	P50	P75	P95
Grains	(g)	231.8	386.0	485.9	574.4	764.0	287.8	414.3	490.8	563.7	719.5	314.7	424.9	496.2	561.6	698.2	350.1	436.0	500.2	552.8	664.3
Potatoes	(g)	0.0[a]	14.3	22.9	36.2	77.7	0.7	15.0	24.2	39.0	73.8	4.1	15.9	25.7	40.3	72.1	6.6	17.9	28.1	44.3	74.8
Sugar and sweetners	(g)	0.0[a]	0.0	0.7	2.0	8.0	0.0[a]	0.1	0.6	2.2	7.9	0.0[a]	0.1	0.6	2.4	7.8	0.0[a]	0.1	0.5	2.4	7.6
Legumes	(g)	8.2	49.5	72.7	103.9	224.6	14.3	50.1	74.0	106.8	205.1	19.4	50.8	74.4	104.7	190.7	21.5	51.2	74.9	107.4	199.0
Nuts and seeds	(g)	0.0[a]	0.2	1.2	2.0	7.6	0.0[a]	0.3	1.1	2.1	8.1	0.0[a]	0.4	1.1	2.0	7.7	0.0[a]	0.4	1.1	2.0	8.6
Vegetables	(g)	36.2	142.4	200.4	292.4	648.1	58.6	145.6	204.2	283.6	467.3	73.5	153.2	209.9	280.4	431.4	93.8	169.7	225.4	291.9	411.9
Fruits	(g)	0.0[a]	146.5	230.6	400.5	724.0	12.7	140.7	228.7	352.2	611.4	33.3	142.0	216.8	306.9	490.6	61.4	151.4	209.1	269.7	372.4
Mushrooms	(g)	0.0[a]	4.5	7.6	13.5	30.7	0.0[a]	4.7	8.4	15.0	30.9	0.4	4.9	9.0	15.5	30.3	1.0	5.3	9.9	17.2	31.4
Seaweed	(g)	0.0[a]	5.5	8.7	14.2	29.8	0.7	5.6	9.3	14.9	27.8	1.6	5.8	9.6	15.3	26.3	2.1	6.3	10.3	15.9	26.8
Fish and shellfish	(g)	10.4	57.0	80.5	114.7	223.0	18.8	58.3	81.9	112.3	182.7	23.8	59.7	82.6	110.8	170.3	28.0	62.9	85.9	111.9	160.1
Meat	(g)	0.0[a]	31.2	52.4	75.4	145.3	2.4	33.6	52.3	75.5	135.0	10.4	36.4	53.9	75.3	128.5	15.8	38.5	55.2	75.9	122.4
Eggs	(g)	0.0[a]	12.3	21.2	34.9	60.6	0.8	13.1	23.1	38.0	57.8	2.7	14.2	25.4	40.5	56.6	4.5	15.6	27.1	42.0	54.7
Dairy products	(g)	0.0[a]	75.3	151.7	302.7	698.0	0.0[a]	77.1	167.7	302.6	648.4	0.0[a]	100.0	190.3	285.6	534.3	26.1	116.5	183.7	242.8	312.9
Fat and oil	(g)	3.9	9.1	11.7	15.2	24.3	4.8	9.2	11.8	15.0	21.6	5.9	9.6	12.0	15.0	20.8	7.1	10.3	12.6	15.5	21.0
Confectionaries	(g)	0.0[a]	7.5	16.9	25.6	60.4	0.0[a]	9.4	17.6	27.5	63.6	0.0[a]	10.3	17.8	28.0	63.4	1.9	11.1	18.3	29.9	67.4
Alcohol beverages	(g)	0.0[a]	0.0[a]	6.0	13.0	162.1	0.0[a]	0.0[a]	4.0	10.3	149.9	0.0[a]	0.0[a]	3.4	9.0	158.4	0.0[a]	0.0[a]	2.8	7.7	160.0
Non-alcohol beverages	(g)	93.2	386.7	667.6	1,100.2	1,868.2	144.5	428.7	685.0	1,062.3	1,787.9	177.4	447.8	699.0	1,049.1	1,771.4	204.7	458.5	711.1	1,037.1	1,714.1
Seasonings	(g)	1.8	6.5	8.7	11.9	21.0	2.3	6.4	8.8	12.0	19.5	2.9	6.5	8.8	11.9	19.0	3.4	6.6	9.0	12.1	18.3
Protein	(g)	51.5	63.3	69.7	77.9	97.9	53.7	64.2	70.0	76.9	90.1	55.7	64.7	70.3	76.3	86.7	55.7	65.2	70.5	75.8	83.7
Total fat	(g)	31.5	48.3	56.5	66.0	84.7	35.2	49.2	56.9	65.6	81.5	38.6	50.5	57.6	65.3	78.9	41.7	51.8	58.0	64.7	76.8
Carbohydrate	(g)	172.5	231.2	259.1	283.7	331.9	187.5	232.9	257.8	280.6	320.0	196.3	235.0	255.8	276.0	309.2	205.8	237.2	254.9	271.3	299.5
Sodium	(mg)	2,773	3,992	4,864	6,030	8,413	2,643	3,899	4,738	5,724	7,477	2,678	3,808	4,586	5,419	6,860	2,691	3,709	4,371	5,069	6,175
Pottasium	(mg)	1,762	2,471	2,880	3,466	4,702	1,878	2,501	2,880	3,298	4,063	1,926	2,503	2,854	3,233	3,860	1,989	2,529	2,847	3,172	3,700
Calcium	(mg)	208	418	521	703	1,105	244	419	537	681	1,018	270	433	545	655	909	291	440	530	612	733
Magnecium	(mg)	213	276	314	370	500	218	277	315	361	451	222	276	314	354	431	223	278	313	351	421
Iron	(mg)	5.9	7.8	8.9	10.5	14.0	6.0	7.8	9.0	10.3	12.9	6.2	7.9	9.0	10.2	12.4	6.5	8.0	9.0	10.2	12.2
Retinol	(µg)	0[a]	214	381	625	1,422	0[a]	226	387	655	1,344	16	232	394	650	1,304	48	218	375	639	1,225
α-carotene	(µg)	0[a]	335	562	949	2,565	0[a]	336	576	956	1,854	65	365	626	1,024	1,823	111	408	705	1,092	1,908
β-carotene	(µg)	534	2,304	3,263	4,711	10,549	753	2,307	3,275	4,601	7,992	1,027	2,398	3,375	4,645	7,579	1,294	2,607	3,626	4,855	7,745
Vitamin D	(µg)	1.7	7.3	10.1	14.3	28.5	2.5	7.3	10.3	14.2	23.6	2.9	7.5	10.4	14.1	22.1	3.0	7.6	10.7	14.1	21.2
Vitamin C	(mg)	41	104	146	217	357	49	105	145	197	294	57	106	142	184	259	71	113	143	176	231
Saturated fat	(g)	6.7	13.5	17.0	21.2	30.1	8.7	14.0	17.4	21.3	28.8	10.2	14.7	17.6	21.0	27.7	11.4	15.1	17.5	20.1	25.0
Dietary fiber	(g)	6.3	10.7	13.2	17.2	26.6	6.6	10.9	13.5	16.5	22.0	7.3	11.0	13.3	15.9	20.4	8.2	11.4	13.5	15.8	19.5

Values: 5, 25, 50, 75, 95 percentile

All intake values were energy-adjusted using residual method.

[a] Negative values were replaced with 0.

eTable 4. Multivariable adjusted hazard ratios and 95% confidence intervals of mortality according to energy-adjusted score for adherence to Healthy Meal stratified by alcohol consumption in men

	Consumed <300 g ethanol equivalent/week (n=27,365)						Consumed ≧300 g ethanol equivalent/week (n=12,425)						
	Quartile of energy-adjusted score for adherence to Healthy Meal						Quartile of energy-adjusted score for adherence to Healthy Meal						
	Q1 (low) (n=6,655)	Q2 (n=6,797)	Q3 (n=6,888)	Q4 (high) (n=7,025)	*P* for trend	1 point increment	Q1 (low) (n=3,249)	Q2 (n=3,161)	Q3 (n=3,066)	Q4 (high) (n=2,949)	*P* for trend	1 point increment	*P* for interaction
Person years	121,056	123,702	126,648	128,647	-	-	58,541	58,187	57,357	54,705	-	-	-
All-cause mortality													
Number of deaths	2,199	2,118	1,963	1,949	-	-	1,161	1,021	894	859	-	-	-
Model 1[a]	1.00 (ref)	0.96 (0.90 −1.02)	0.85 (0.80 −0.91)	0.82 (0.77 −0.87)	<0.001	0.90 (0.87 −0.92)	1.00 (ref)	0.89 (0.82 −0.97)	0.76 (0.70 −0.83)	0.75 (0.69 −0.82)	<0.001	0.86 (0.83 −0.90)	-
Model 2[b]	1.00 (ref)	1.00 (0.94 −1.06)	0.90 (0.85 −0.96)	0.87 (0.82 −0.93)	<0.001	0.93 (0.90 −0.96)	1.00 (ref)	0.94 (0.87 −1.03)	0.82 (0.75 −0.90)	0.84 (0.77 −0.92)	<0.001	0.92 (0.88 −0.95)	-
Model 3[c]	1.00 (ref)	1.00 (0.94 −1.06)	0.90 (0.85 −0.96)	0.87 (0.81 −0.92)	<0.001	0.93 (0.90 −0.96)	1.00 (ref)	0.94 (0.86 −1.02)	0.82 (0.75 −0.90)	0.84 (0.77 −0.92)	<0.001	0.92 (0.88 −0.95)	0.389
Cancer mortality													
Number of deaths	805	780	738	750	-	-	474	426	362	381	-	-	-
Model 1[a]	1.00 (ref)	0.96 (0.87 −1.06)	0.88 (0.79 −0.97)	0.86 (0.78 −0.95)	<0.001	0.92 (0.88 −0.96)	1.00 (ref)	0.90 (0.79 −1.03)	0.76 (0.67 −0.88)	0.82 (0.72 −0.94)	0.0005	0.89 (0.84 −0.95)	-
Model 2[b]	1.00 (ref)	0.99 (0.90 −1.10)	0.93 (0.84 −1.03)	0.92 (0.83 −1.02)	0.0555	0.95 (0.91 −0.999)	1.00 (ref)	0.94 (0.82 −1.07)	0.80 (0.70 −0.92)	0.90 (0.78 −1.03)	0.0315	0.93 (0.88 −0.996)	-
Model 3[c]	1.00 (ref)	0.99 (0.90 −1.10)	0.93 (0.84 −1.02)	0.92 (0.83 −1.02)	0.0519	0.95 (0.91 −0.999)	1.00 (ref)	0.94 (0.82 −1.07)	0.80 (0.70 −0.92)	0.90 (0.78 −1.03)	0.0312	0.93 (0.88 −0.996)	0.660
Cardiovascular disease mortality													
Number of deaths	578	529	490	488	-	-	300	245	222	196	-	-	-
Model 1[a]	1.00 (ref)	0.92 (0.82 −1.03)	0.81 (0.72 −0.92)	0.78 (0.69 −0.88)	<0.001	0.88 (0.84 −0.93)	1.00 (ref)	0.82 (0.69 −0.97)	0.73 (0.61 −0.86)	0.66 (0.55 −0.79)	<0.001	0.82 (0.76 −0.89)	-
Model 2[b]	1.00 (ref)	0.97 (0.86 −1.10)	0.88 (0.78 −0.99)	0.85 (0.75 −0.97)	0.0042	0.93 (0.88 −0.98)	1.00 (ref)	0.88 (0.74 −1.04)	0.79 (0.66 −0.94)	0.74 (0.61 −0.89)	0.0005	0.87 (0.80 −0.95)	-
Model 3[c]	1.00 (ref)	0.97 (0.86 −1.10)	0.88 (0.78 −0.99)	0.85 (0.75 −0.97)	0.0041	0.93 (0.88 −0.98)	1.00 (ref)	0.87 (0.73 −1.03)	0.78 (0.66 −0.94)	0.73 (0.61 −0.88)	<0.001	0.87 (0.80 −0.94)	0.198
Heart disease mortality													
Number of deaths	306	289	261	267	-	-	145	121	104	109	-	-	-
Model 1[a]	1.00 (ref)	0.95 (0.81 −1.11)	0.82 (0.69 −0.97)	0.81 (0.69 −0.96)	0.0036	0.89 (0.83 −0.96)	1.00 (ref)	0.83 (0.65 −1.06)	0.69 (0.54 −0.89)	0.75 (0.58 −0.96)	0.0073	0.89 (0.79 −0.99)	-
Model 2[b]	1.00 (ref)	1.01 (0.85 −1.18)	0.88 (0.75 −1.04)	0.88 (0.74 −1.04)	0.0648	0.94 (0.87 −1.01)	1.00 (ref)	0.87 (0.68 −1.11)	0.74 (0.57 −0.96)	0.82 (0.64 −1.06)	0.0617	0.94 (0.83 −1.06)	-
Model 3[c]	1.00 (ref)	1.00 (0.85 −1.18)	0.88 (0.74 −1.04)	0.88 (0.74 −1.04)	0.0578	0.93 (0.86 −1.01)	1.00 (ref)	0.87 (0.68 −1.11)	0.74 (0.57 −0.96)	0.82 (0.64 −1.06)	0.0639	0.94 (0.84 −1.06)	0.762
Cerebrovascular disease mortality													
Number of deaths	216	186	182	175	-	-	134	110	97	67	-	-	-
Model 1[a]	1.00 (ref)	0.86 (0.71 −1.05)	0.81 (0.66 −0.98)	0.75 (0.61 −0.91)	0.0032	0.87 (0.79 −0.95)	1.00 (ref)	0.83 (0.65 −1.07)	0.72 (0.55 −0.94)	0.51 (0.38 −0.69)	<0.001	0.71 (0.63 −0.80)	-
Model 2[b]	1.00 (ref)	0.91 (0.75 −1.11)	0.87 (0.71 −1.07)	0.81 (0.66 −0.999)	0.0453	0.91 (0.83 −1.00)	1.00 (ref)	0.91 (0.70 −1.18)	0.80 (0.61 −1.05)	0.59 (0.44 −0.80)	0.0005	0.77 (0.68 −0.87)	-
Model 3[c]	1.00 (ref)	0.91 (0.75 −1.11)	0.88 (0.72 −1.07)	0.82 (0.67 −1.01)	0.0515	0.91 (0.83 −1.01)	1.00 (ref)	0.90 (0.70 −1.17)	0.80 (0.61 −1.04)	0.59 (0.44 −0.79)	0.0005	0.76 (0.68 −0.87)	0.012
Respiratory disease mortality													
Number of deaths	205	219	195	172	-	-	81	89	61	63	-	-	-
Model 1[a]	1.00 (ref)	1.06 (0.87 −1.28)	0.89 (0.73 −1.08)	0.75 (0.61 −0.92)	0.0016	0.88 (0.80 −0.96)	1.00 (ref)	1.11 (0.82 −1.51)	0.71 (0.51 −0.99)	0.77 (0.56 −1.08)	0.0213	0.86 (0.75 −1.000)	-
Model 2[b]	1.00 (ref)	1.10 (0.91 −1.33)	0.95 (0.78 −1.16)	0.80 (0.65 −0.98)	0.0131	0.91 (0.83 −0.997)	1.00 (ref)	1.24 (0.92 −1.69)	0.83 (0.59 −1.16)	0.97 (0.69 −1.36)	0.3538	0.98 (0.84 −1.14)	-
Model 3[c]	1.00 (ref)	1.10 (0.91 −1.33)	0.95 (0.78 −1.16)	0.79 (0.64 −0.98)	0.0121	0.91 (0.83 −0.996)	1.00 (ref)	1.25 (0.92 −1.70)	0.83 (0.59 −1.17)	0.97 (0.69 −1.37)	0.3697	0.98 (0.84 −1.14)	0.752

Adjusted hazard ratios and 95% confidence intervals. Analysis using Cox proportional hazards model.

[a] Model 1 was adjusted for age and study area (11 areas).

[b] Model 2 was adjusted as for model 1 plus body mass index, smoking status, total physical activity, history or medication of hypertension/ diabetes/ dyslipidemia, occupation, green tea consumption and coffee consumption.

[c] Model 3 was adjusted as for model 2 plus energy intake.

eTable 5. Multivariable adjusted hazard ratios and 95% confidence intervals of mortality according to energy-adjusted score for adherence to Healthy Meal stratified by alcohol consumption in women

	Consumed <300 g ethanol equivalent/week (n=45,502)					
	Quartile of energy-adjusted score for adherence to Healthy Meal					1 point increment
	Q1 (low) (n=6,655)	Q2 (n=6,797)	Q3 (n=6,888)	Q4 (high) (n=7,025)	*P* for trend	
Person years	219,658	223,338	224,327	223,775	-	-
All-cause mortality						
Number of deaths	2,225	2,023	1,856	1,721	-	-
Model 1[a]	1.00 (ref)	0.97 (0.92 −1.03)	0.91 (0.86 −0.97)	0.89 (0.83 −0.94)	<0.001	0.94 (0.83 −0.94)
Model 2[b]	1.00 (ref)	1.00 (0.94 −1.06)	0.96 (0.90 −1.02)	0.94 (0.88 −0.998)	<0.001	0.97 (0.88 −0.998)
Model 3[c]	1.00 (ref)	1.00 (0.94 −1.06)	0.95 (0.90 −1.02)	0.93 (0.88 −0.996)	0.018	0.97 (0.88 −0.996)
Cancer mortality						
Number of deaths	686	705	627	619	-	-
Model 1[a]	1.00 (ref)	1.06 (0.96 −1.18)	0.96 (0.86 −1.07)	0.98 (0.88 −1.10)	0.390	0.99 (0.94 −1.10)
Model 2[b]	1.00 (ref)	1.08 (0.97 −1.20)	0.98 (0.88 −1.10)	1.01 (0.90 −1.12)	0.679	1.00 (0.95 −1.12)
Model 3[c]	1.00 (ref)	1.08 (0.97 −1.20)	0.98 (0.88 −1.10)	1.01 (0.90 −1.12)	0.680	1.00 (0.95 −1.12)
Cardiovascular disease mortality						
Number of deaths	635	559	521	494	-	-
Model 1[a]	1.00 (ref)	0.96 (0.85 −1.07)	0.91 (0.81 −1.03)	0.92 (0.81 −1.03)	0.095	0.95 (0.90 −1.01)
Model 2[b]	1.00 (ref)	0.99 (0.88 −1.11)	0.97 (0.86 −1.09)	0.98 (0.87 −1.11)	0.666	0.99 (0.94 −1.05)
Model 3[c]	1.00 (ref)	0.99 (0.88 −1.11)	0.97 (0.86 −1.09)	0.98 (0.87 −1.10)	0.642	0.99 (0.94 −1.05)
Heart disease mortality						
Number of deaths	319	304	254	270	-	-
Model 1[a]	1.00 (ref)	1.05 (0.90 −1.23)	0.89 (0.76 −1.06)	1.00 (0.85 −1.18)	0.560	0.99 (0.92 −1.07)
Model 2[b]	1.00 (ref)	1.10 (0.94 −1.29)	0.96 (0.82 −1.14)	1.10 (0.93 −1.30)	0.555	1.04 (0.96 −1.13)
Model 3[c]	1.00 (ref)	1.10 (0.94 −1.29)	0.97 (0.82 −1.14)	1.10 (0.93 −1.30)	0.551	1.04 (0.97 −1.13)
Cerebrovascular disease mortality						
Number of deaths	260	211	221	168	-	-
Model 1[a]	1.00 (ref)	0.87 (0.72 −1.04)	0.94 (0.78 −1.12)	0.76 (0.62 −0.92)	0.016	0.88 (0.81 −0.96)
Model 2[b]	1.00 (ref)	0.89 (0.74 −1.06)	0.98 (0.81 −1.17)	0.79 (0.65 −0.96)	0.062	0.90 (0.83 −0.99)
Model 3[c]	1.00 (ref)	0.88 (0.74 −1.06)	0.97 (0.81 −1.17)	0.79 (0.65 −0.96)	0.055	0.90 (0.82 −0.99)
Respiratory disease mortality						
Number of deaths	180	151	132	92	-	-
Model 1[a]	1.00 (ref)	0.94 (0.76 −1.16)	0.83 (0.66 −1.04)	0.63 (0.49 −0.80)	<0.001	0.81 (0.73 −0.90)
Model 2[b]	1.00 (ref)	0.99 (0.80 −1.23)	0.91 (0.73 −1.15)	0.70 (0.54 −0.90)	0.007	0.86 (0.77 −0.96)
Model 3[c]	1.00 (ref)	0.99 (0.80 −1.23)	0.91 (0.73 −1.15)	0.70 (0.54 −0.90)	0.007	0.86 (0.77 −0.96)

Adjusted hazard ratios and 95% confidence intervals. Analysis using Cox proportional hazards model.

Since the number of women who consumed more than 300 g of ethanol was very small (n=531), only those who consumed less than 300 g were analyzed.

a Model1 was adjusted for age and study area (11 areas).

b Model2 was adjusted as for model1 plus body mass index, smoking status, total physical activity, history or medication of hypertension/ diabetes/ dyslipidemia, occupation, green tea consumption and coffee consumption.

eTable 6. Multivariable adjusted hazard ratios and 95% confidence intervals of mortality according to energy-adjusted score for adherence to Healthy Meal stratified by smoking status in men

	Non-smoker/ former smoker (n=20,214)						Current smoker (n=18,015)						
	Quartile of energy-adjusted score for adherence to Healthy Meal					1 point increment	Quartile of energy-adjusted score for adherence to Healthy Meal					1 point increment	*P* for interaction
	Q1 (low) (n=4,445)	Q2 (n=4,871)	Q3 (n=5,227)	Q4 (high) (n=5,671)	*P* for trend		Q1 (low) (n=4,980)	Q2 (n=4,703)	Q3 (n=4,366)	Q4 (high) (n=3,966)	*P* for trend		
Person years	82,765	90,856	97,979	104,976	-	-	89,240	84,764	80,025	72,685	-	-	-
All-cause mortality													
Number of deaths	1,324	1,332	1,342	1,486	-	-	1,802	1,633	1,368	1,180	-	-	-
Model 1[a]	1.00 (ref)	0.93 (0.86 −1.00)	0.85 (0.79 −0.91)	0.88 (0.81 −0.94)	<0.001	0.92 (0.89 −0.95)	1.00 (ref)	0.97 (0.91 −1.04)	0.85 (0.79 −0.91)	0.79 (0.73 −0.85)	<0.001	0.89 (0.86 −0.92)	-
Model 2[b]	1.00 (ref)	0.94 (0.87 −1.02)	0.87 (0.81 −0.94)	0.89 (0.82 −0.96)	<0.001	0.93 (0.90 −0.96)	1.00 (ref)	1.00 (0.94 −1.07)	0.88 (0.82 −0.95)	0.82 (0.76 −0.89)	<0.001	0.92 (0.89 −0.95)	-
Model 3[c]	1.00 (ref)	0.94 (0.87 −1.02)	0.87 (0.81 −0.94)	0.89 (0.82 −0.96)	<0.001	0.93 (0.90 −0.96)	1.00 (ref)	1.00 (0.94 −1.07)	0.88 (0.82 −0.95)	0.82 (0.76 −0.89)	<0.001	0.92 (0.89 −0.95)	0.375
Cancer mortality													
Number of deaths	462	457	480	550	-	-	722	679	577	519	-	-	-
Model 1[a]	1.00 (ref)	0.91 (0.80 −1.04)	0.87 (0.77 −0.99)	0.93 (0.82 −1.05)	0.242	0.94 (0.88 −0.99)	1.00 (ref)	1.00 (0.90 −1.11)	0.89 (0.80 −1.00)	0.86 (0.77 −0.97)	0.002	0.94 (0.89 −0.99)	-
Model 2[b]	1.00 (ref)	0.92 (0.80 −1.04)	0.88 (0.77 −1.00)	0.94 (0.83 −1.06)	0.295	0.94 (0.88 −0.99)	1.00 (ref)	1.02 (0.91 −1.13)	0.91 (0.81 −1.02)	0.89 (0.79 −1.00)	0.013	0.96 (0.91 −1.01)	-
Model 3[c]	1.00 (ref)	0.92 (0.80 −1.04)	0.88 (0.77 −1.00)	0.94 (0.83 −1.06)	0.292	0.94 (0.88 −0.99)	1.00 (ref)	1.02 (0.91 −1.13)	0.91 (0.81 −1.02)	0.89 (0.79 −1.00)	0.013	0.96 (0.91 −1.01)	0.773
Cardiovascular disease mortality													
Number of deaths	352	363	338	360	-	-	482	379	322	286	-	-	-
Model 1[a]	1.00 (ref)	0.95 (0.82 −1.10)	0.80 (0.69 −0.93)	0.80 (0.69 −0.92)	<0.001	0.88 (0.82 −0.94)	1.00 (ref)	0.85 (0.74 −0.97)	0.75 (0.65 −0.86)	0.72 (0.62 −0.83)	<0.001	0.85 (0.79 −0.90)	-
Model 2[b]	1.00 (ref)	0.98 (0.84 −1.13)	0.84 (0.72 −0.97)	0.82 (0.71 −0.96)	0.002	0.90 (0.84 −0.96)	1.00 (ref)	0.89 (0.77 −1.02)	0.78 (0.68 −0.90)	0.75 (0.65 −0.87)	<0.001	0.87 (0.82 −0.94)	-
Model 3[c]	1.00 (ref)	0.98 (0.85 −1.14)	0.85 (0.73 −0.98)	0.83 (0.72 −0.97)	0.004	0.91 (0.85 −0.97)	1.00 (ref)	0.89 (0.78 −1.02)	0.78 (0.68 −0.90)	0.75 (0.65 −0.87)	<0.001	0.87 (0.82 −0.94)	0.367
Heart disease mortality													
Number of deaths	180	200	183	201	-	-	257	188	157	159	-	-	-
Model 1[a]	1.00 (ref)	1.03 (0.84 −1.26)	0.85 (0.69 −1.04)	0.88 (0.72 −1.07)	0.069	0.92 (0.84 −1.01)	1.00 (ref)	0.79 (0.65 −0.95)	0.69 (0.56 −0.84)	0.75 (0.62 −0.91)	0.001	0.87 (0.79 −0.95)	-
Model 2[b]	1.00 (ref)	1.05 (0.86 −1.29)	0.88 (0.71 −1.08)	0.89 (0.72 −1.09)	0.104	0.93 (0.85 −1.03)	1.00 (ref)	0.82 (0.68 −0.99)	0.72 (0.59 −0.88)	0.79 (0.64 −0.96)	0.005	0.89 (0.81 −0.98)	-
Model 3[c]	1.00 (ref)	1.05 (0.86 −1.29)	0.88 (0.72 −1.09)	0.89 (0.72 −1.10)	0.111	0.93 (0.85 −1.03)	1.00 (ref)	0.82 (0.68 −0.99)	0.72 (0.59 −0.88)	0.79 (0.64 −0.96)	0.006	0.89 (0.81 −0.98)	0.375
Cerebrovascular disease mortality													
Number of deaths	141	136	127	126	-	-	186	150	128	99	-	-	-
Model 1[a]	1.00 (ref)	0.89 (0.70 −1.13)	0.75 (0.59 −0.96)	0.69 (0.54 −0.88)	0.001	0.81 (0.73 −0.90)	1.00 (ref)	1.28 (1.02 −1.61)	0.91 (0.71 −1.17)	0.81 (0.62 −1.05)	0.027	0.90 (0.81 −1.01)	-
Model 2[b]	1.00 (ref)	0.92 (0.72 −1.17)	0.80 (0.62 −1.02)	0.73 (0.57 −0.93)	0.006	0.83 (0.75 −0.93)	1.00 (ref)	1.32 (1.05 −1.67)	0.95 (0.74 −1.22)	0.86 (0.65 −1.12)	0.081	0.93 (0.83 −1.05)	-
Model 3[c]	1.00 (ref)	0.93 (0.73 −1.18)	0.81 (0.64 −1.04)	0.75 (0.58 −0.96)	0.012	0.85 (0.76 −0.95)	1.00 (ref)	1.32 (1.05 −1.66)	0.95 (0.74 −1.22)	0.86 (0.65 −1.12)	0.081	0.93 (0.83 −1.05)	0.801
Respiratory disease mortality													
Number of deaths	129	127	134	138	-	-	138	160	111	93	-	-	-
Model 1[a]	1.00 (ref)	0.89 (0.69 −1.13)	0.84 (0.66 −1.07)	0.81 (0.64 −1.03)	0.082	0.92 (0.82 −1.03)	1.00 (ref)	1.11 (0.82 −1.51)	0.71 (0.51 −0.99)	0.77 (0.56 −1.08)	0.021	0.86 (0.75 −1.00)	-
Model 2[b]	1.00 (ref)	0.93 (0.73 −1.19)	0.89 (0.70 −1.14)	0.83 (0.65 −1.07)	0.140	0.94 (0.84 −1.06)	1.00 (ref)	1.24 (0.92 −1.69)	0.83 (0.59 −1.16)	0.97 (0.69 −1.36)	0.354	0.98 (0.84 −1.14)	-
Model 3[c]	1.00 (ref)	0.93 (0.73 −1.19)	0.89 (0.69 −1.13)	0.83 (0.65 −1.06)	0.126	0.94 (0.84 −1.05)	1.00 (ref)	1.25 (0.92 −1.70)	0.83 (0.59 −1.17)	0.97 (0.69 −1.37)	0.370	0.98 (0.84 −1.14)	0.887

Adjusted hazard ratios and 95% confidence intervals. Analysis using Cox proportional hazards model.

[a] Model 1 was adjusted for age and study area (11 areas).

[b] Model 2 was adjusted as for model 1 plus body mass index, alcohol drinking, total physical activity, history or medication of hypertension/ diabetes/ dyslipidemia, occupation, green tea consumption and coffee consumption.

[c] Model 3 was adjusted as for model 2 plus energy intake.

eTable 7. Multivariable adjusted hazard ratios and 95% confidence intervals of mortality according to energy-adjusted score for adherence to Healthy Meal stratified by smoking status in women

	Non-smoker/ former smoker (n=41,834)						Current smoker (n=2,584)						
	Quartile of energy-adjusted score for adherence to Healthy Meal					1 point increment	Quartile of energy-adjusted score for adherence to Healthy Meal					1 point increment	*P* for interaction
	Q1 (low) (n=10,074)	Q2 (n=10,454)	Q3 (n=10,611)	Q4 (high) (n=10,695)	*P* for trend		Q1 (low) (n=780)	Q2 (n=679)	Q3 (n=574)	Q4 (high) (n=551)	*P* for trend		
Person years	199,735	206,743	209,655	209,741	-	-	13,942	12,451	10,153	10,144	-	-	-
All-cause mortality													
Number of deaths	1,881	1,802	1,661	1,552	-	-	192	115	114	86	-	-	-
Model 1[a]	1.00 (ref)	0.99 (0.93 −1.06)	0.93 (0.87 −0.99)	0.90 (0.84 −0.97)	<0.001	0.95 (0.92 −0.98)	1.00 (ref)	0.71 (0.56 −0.89)	0.87 (0.69 −1.10)	0.71 (0.55 −0.91)	0.023	0.81 (0.72 −0.92)	-
Model 2[b]	1.00 (ref)	1.01 (0.95 −1.08)	0.96 (0.90 −1.03)	0.94 (0.88 −1.01)	0.036	0.97 (0.94 −1.01)	1.00 (ref)	0.72 (0.57 −0.92)	0.95 (0.74 −1.20)	0.76 (0.58 −0.99)	0.130	0.85 (0.75 −0.96)	-
Model 3[c]	1.00 (ref)	1.01 (0.95 −1.08)	0.96 (0.90 −1.03)	0.94 (0.88 −1.01)	0.033	0.97 (0.94 −1.00)	1.00 (ref)	0.74 (0.58 −0.94)	0.98 (0.77 −1.25)	0.78 (0.60 −1.03)	0.229	0.87 (0.77 −0.98)	0.066
Cancer mortality													
Number of deaths	596	628	560	554	-	-	55	44	52	41	-	-	-
Model 1[a]	1.00 (ref)	1.06 (0.95 −1.19)	0.96 (0.85 −1.08)	0.98 (0.87 −1.10)	0.337	0.99 (0.94 −1.05)	1.00 (ref)	0.95 (0.64 −1.42)	1.41 (0.96 −2.06)	1.16 (0.77 −1.74)	0.188	1.09 (0.89 −1.32)	-
Model 2[b]	1.00 (ref)	1.07 (0.96 −1.20)	0.97 (0.86 −1.09)	0.99 (0.88 −1.11)	0.462	1.00 (0.94 −1.05)	1.00 (ref)	0.95 (0.63 −1.42)	1.47 (0.99 −2.17)	1.19 (0.79 −1.81)	0.137	1.10 (0.90 −1.34)	-
Model 3[c]	1.00 (ref)	1.07 (0.96 −1.20)	0.97 (0.86 −1.09)	0.99 (0.88 −1.11)	0.479	1.00 (0.94 −1.06)	1.00 (ref)	0.97 (0.65 −1.46)	1.54 (1.04 −2.29)	1.25 (0.82 −1.92)	0.086	1.13 (0.92 −1.39)	0.453
Cardiovascular disease mortality													
Number of deaths	530	487	461	448	-	-	68	35	30	22	-	-	-
Model 1[a]	1.00 (ref)	0.96 (0.85 −1.09)	0.93 (0.82 −1.05)	0.95 (0.83 −1.07)	0.310	0.97 (0.91 −1.03)	1.00 (ref)	0.59 (0.39 −0.89)	0.63 (0.41 −0.96)	0.50 (0.31 −0.81)	0.003	0.68 (0.55 −0.84)	-
Model 2[b]	1.00 (ref)	0.98 (0.87 −1.11)	0.97 (0.85 −1.10)	0.99 (0.88 −1.13)	0.856	0.99 (0.94 −1.06)	1.00 (ref)	0.63 (0.41 −0.96)	0.64 (0.41 −1.00)	0.57 (0.34 −0.94)	0.015	0.71 (0.57 −0.88)	-
Model 3[c]	1.00 (ref)	0.98 (0.87 −1.11)	0.97 (0.85 −1.10)	0.99 (0.87 −1.13)	0.823	0.99 (0.93 −1.06)	1.00 (ref)	0.63 (0.41 −0.96)	0.64 (0.41 −1.00)	0.57 (0.34 −0.93)	0.015	0.70 (0.56 −0.88)	0.014
Heart disease mortality													
Number of deaths	262	261	224	244	-	-	37	17	10	13	-	-	-
Model 1[a]	1.00 (ref)	1.05 (0.89 - 1.25)	0.92 (0.77 - 1.10)	1.05 (0.88 - 1.25)	0.993	1.02 (0.94 - 1.11)	1.00 (ref)	0.54 (0.30 −0.96)	0.39 (0.20 −0.80)	0.56 (0.30 −1.07)	0.019	0.62 (0.46 −0.84)	-
Model 2[b]	1.00 (ref)	1.08 (0.91 - 1.29)	0.96 (0.80 - 1.15)	1.11 (0.93 - 1.33)	0.498	1.05 (0.97 - 1.15)	1.00 (ref)	0.57 (0.31 −1.05)	0.42 (0.20 −0.88)	0.67 (0.34 −1.30)	0.075	0.65 (0.47 −0.90)	-
Model 3[c]	1.00 (ref)	1.09 (0.91 - 1.29)	0.96 (0.80 - 1.15)	1.11 (0.93 - 1.33)	0.499	1.05 (0.97 - 1.15)	1.00 (ref)	0.57 (0.31 −1.05)	0.43 (0.21 −0.89)	0.67 (0.34 −1.32)	0.082	0.66 (0.47 −0.91)	0.010
Cerebrovascular disease mortality													
Number of deaths	219	184	198	156	-	-	24	15	13	7	-	-	-
Model 1[a]	1.00 (ref)	0.87 (0.71 −1.06)	0.96 (0.79 −1.16)	0.80 (0.65 −0.98)	0.078	0.90 (0.81 −0.99)	1.00 (ref)	0.72 (0.38 −1.38)	0.75 (0.38 −1.48)	0.43 (0.18 −0.999)	0.058	0.69 (0.49 −0.96)	-
Model 2[b]	1.00 (ref)	0.88 (0.73 −1.08)	0.99 (0.81 −1.20)	0.83 (0.67 −1.02)	0.182	0.92 (0.83 −1.01)	1.00 (ref)	0.75 (0.38 −1.46)	0.73 (0.36 −1.48)	0.47 (0.19 −1.12)	0.090	0.71 (0.50 −1.01)	-
Model 3[c]	1.00 (ref)	0.88 (0.72 −1.07)	0.98 (0.81 −1.19)	0.82 (0.67 −1.01)	0.161	0.91 (0.83 −1.01)	1.00 (ref)	0.74 (0.38 −1.45)	0.73 (0.36 −1.47)	0.46 (0.19 −1.11)	0.085	0.70 (0.49 −1.00)	0.266
Respiratory disease mortality													
Number of deaths	149	136	119	74	-	-	13	8	5	5	-	-	-
Model 1[a]	1.00 (ref)	0.97 (0.77 −1.23)	0.86 (0.67 −1.09)	0.58 (0.44 −0.76)	<0.001	0.81 (0.72 −0.91)	1.00 (ref)	0.78 (0.32 −1.92)	0.65 (0.23 −1.86)	0.84 (0.29 −2.41)	0.572	0.72 (0.43 −1.20)	-
Model 2[b]	1.00 (ref)	1.02 (0.81 −1.29)	0.93 (0.73 −1.19)	0.63 (0.47 −0.83)	0.002	0.85 (0.75 −0.96)	1.00 (ref)	0.97 (0.36 −2.64)	0.94 (0.30 −2.92)	1.39 (0.43 −4.49)	0.696	0.93 (0.53 −1.65)	-
Model 3[c]	1.00 (ref)	1.02 (0.81 −1.29)	0.93 (0.73 −1.19)	0.63 (0.47 −0.83)	0.002	0.85 (0.75 −0.96)	1.00 (ref)	0.99 (0.36 −2.70)	1.01 (0.32 −3.19)	1.44 (0.44 −4.70)	0.622	0.97 (0.54 −1.74)	0.850

Adjusted hazard ratios and 95% confidence intervals. Analysis using Cox proportional hazards model.

[a] Model 1 was adjusted for age and study area (11 areas).

[b] Model 2 was adjusted as for model 1 plus body mass index, alcohol drinking, total physical activity, history or medication of hypertension/ diabetes/ dyslipidemia, occupation, green tea consumption and coffee consumption.

[c] Model 3 was adjusted as for model 2 plus energy intake.

eTable 8. Multivariable adjusted hazard ratios and 95% confidence intervals of mortality according to energy-adjusted score for adherence to Healthy Meal stratified by occupation in men

	Agriculture/ Fishery/ Forestry (n=10,280)						Others (n=29,942)						*P* for interaction
	Quartile of energy-adjusted score for adherence to Healthy Meal					1 point increment	Quartile of energy-adjusted score for adherence to Healthy Meal					1 point increment	
	Q1 (low) (n=2,744)	Q2 (n=2,612)	Q3 (n=2,517)	Q4 (high) (n=2,407)	*P* for trend		Q1 (low) (n=7,311)	Q2 (n=7,444)	Q3 (n=7,539)	Q4 (high) (n=7,648)	*P* for trend		
Person years	50,631	47,712	47,542	44,421	-	-	131,473	135,842	138,248	140,256	-	-	-
All-cause mortality													
Number of deaths	1,161	1,113	969	940	-	-	2,276	2,072	1,934	1,905	-	-	-
Model 1[a]	1.00 (ref)	1.03 (0.95 −1.12)	0.88 (0.81 −0.96)	0.88 (0.81 −0.96)	<0.001	0.93 (0.89 −0.96)	1.00 (ref)	0.88 (0.83 −0.94)	0.79 (0.75 −0.84)	0.75 (0.71 −0.80)	<0.001	0.87 (0.84 −0.89)	-
Model 2[b]	1.00 (ref)	1.06 (0.97 −1.15)	0.91 (0.83 −0.99)	0.93 (0.85 −1.01)	0.009	0.95 (0.91 −0.99)	1.00 (ref)	0.94 (0.88 −0.996)	0.86 (0.81 −0.92)	0.83 (0.78 −0.89)	<0.001	0.91 (0.89 −0.94)	-
Model 3[c]	1.00 (ref)	1.06 (0.97 −1.15)	0.91 (0.83 −0.99)	0.93 (0.85 −1.01)	0.008	0.95 (0.91 −0.99)	1.00 (ref)	0.94 (0.88 −0.997)	0.86 (0.81 −0.92)	0.83 (0.78 −0.88)	<0.001	0.91 (0.89 −0.94)	0.051
Cancer mortality													
Number of deaths	433	388	354	341	-	-	871	831	764	800	-	-	-
Model 1[a]	1.00 (ref)	0.96 (0.84 −1.10)	0.87 (0.75 −1.00)	0.87 (0.75 −1.00)	0.022	0.93 (0.87 −0.99)	1.00 (ref)	0.92 (0.84 −1.01)	0.82 (0.74 −0.90)	0.82 (0.75 −0.91)	<0.001	0.90 (0.86 −0.94)	-
Model 2[b]	1.00 (ref)	0.98 (0.85 −1.13)	0.89 (0.77 −1.03)	0.92 (0.80 −1.07)	0.148	0.96 (0.89 −1.02)	1.00 (ref)	0.96 (0.87 −1.06)	0.88 (0.80 −0.97)	0.91 (0.82 −0.999)	0.015	0.94 (0.90 −0.99)	-
Model 3[c]	1.00 (ref)	0.98 (0.86 −1.13)	0.89 (0.78 −1.03)	0.93 (0.80 −1.08)	0.173	0.96 (0.90 −1.02)	1.00 (ref)	0.96 (0.88 −1.06)	0.88 (0.80 −0.97)	0.90 (0.82 −0.998)	0.014	0.94 (0.90 −0.99)	0.955
Cardiovascular disease mortality													
Number of deaths	314	305	246	240	-	-	590	483	473	450	-	-	-
Model 1[a]	1.00 (ref)	1.05 (0.90 −1.23)	0.82 (0.69 −0.97)	0.83 (0.70 −0.99)	0.004	0.91 (0.84 −0.98)	1.00 (ref)	0.80 (0.71 −0.90)	0.75 (0.66 −0.85)	0.69 (0.61 −0.77)	<0.001	0.83 (0.79 −0.88)	-
Model 2[b]	1.00 (ref)	1.07 (0.91 −1.25)	0.85 (0.72 −1.01)	0.87 (0.73 −1.03)	0.021	0.93 (0.86 −1.00)	1.00 (ref)	0.86 (0.76 −0.97)	0.82 (0.72 −0.93)	0.76 (0.67 −0.87)	<0.001	0.89 (0.84 −0.94)	-
Model 3[c]	1.00 (ref)	1.07 (0.91 −1.26)	0.86 (0.72 −1.02)	0.88 (0.74 −1.04)	0.028	0.93 (0.86 −1.01)	1.00 (ref)	0.86 (0.76 −0.97)	0.82 (0.72 −0.93)	0.76 (0.67 −0.87)	<0.001	0.89 (0.84 −0.94)	0.120
Heart disease mortality													
Number of deaths	160	161	134	139	-	-	309	255	237	239	-	-	-
Model 1[a]	1.00 (ref)	1.09 (0.88 −1.36)	0.88 (0.70 −1.11)	0.95 (0.76 −1.20)	0.330	0.95 (0.86 −1.06)	1.00 (ref)	0.80 (0.68 −0.95)	0.72 (0.61 −0.85)	0.69 (0.59 −0.82)	<0.001	0.85 (0.79 −0.92)	-
Model 2[b]	1.00 (ref)	1.12 (0.90 −1.39)	0.93 (0.73 −1.17)	1.00 (0.79 −1.27)	0.637	0.98 (0.88 −1.09)	1.00 (ref)	0.86 (0.72 −1.01)	0.77 (0.65 −0.92)	0.76 (0.64 −0.91)	0.001	0.90 (0.83 −0.98)	-
Model 3[c]	1.00 (ref)	1.12 (0.90 −1.39)	0.93 (0.73 −1.17)	1.00 (0.80 −1.27)	0.651	0.98 (0.88 −1.09)	1.00 (ref)	0.86 (0.72 −1.01)	0.77 (0.65 −0.92)	0.76 (0.64 −0.91)	0.001	0.90 (0.83 −0.97)	0.154
Cerebrovascular disease mortality													
Number of deaths	128	119	87	83	-	-	229	183	193	163	-	-	-
Model 1[a]	1.00 (ref)	1.00 (0.78 −1.28)	0.70 (0.54 −0.93)	0.70 (0.53 −0.92)	0.002	0.83 (0.73 −0.94)	1.00 (ref)	0.78 (0.64 −0.94)	0.79 (0.65 −0.95)	0.64 (0.52 −0.78)	<0.001	0.80 (0.73 −0.87)	-
Model 2[b]	1.00 (ref)	1.02 (0.79 −1.31)	0.73 (0.55 −0.96)	0.73 (0.55 −0.97)	0.005	0.85 (0.75 −0.96)	1.00 (ref)	0.85 (0.70 −1.04)	0.88 (0.73 −1.07)	0.73 (0.59 −0.90)	0.007	0.86 (0.78 −0.94)	-
Model 3[c]	1.00 (ref)	1.02 (0.79 −1.31)	0.74 (0.56 −0.97)	0.74 (0.56 −0.98)	0.007	0.85 (0.75 −0.97)	1.00 (ref)	0.85 (0.70 −1.04)	0.88 (0.73 −1.07)	0.73 (0.59 −0.90)	0.007	0.86 (0.78 −0.94)	0.635
Respiratory disease mortality													
Number of deaths	104	127	95	93	-	-	187	189	168	151	-	-	-
Model 1[a]	1.00 (ref)	1.32 (1.02 −1.72)	0.93 (0.70 −1.23)	0.94 (0.71 −1.25)	0.236	0.97 (0.85 −1.10)	1.00 (ref)	0.96 (0.79 −1.18)	0.82 (0.66 −1.00)	0.70 (0.57 −0.87)	<0.001	0.84 (0.77 −0.93)	-
Model 2[b]	1.00 (ref)	1.37 (1.06 −1.78)	0.97 (0.73 −1.29)	1.02 (0.77 −1.36)	0.552	1.01 (0.89 −1.15)	1.00 (ref)	1.05 (0.86 −1.29)	0.90 (0.73 −1.12)	0.79 (0.63 −0.99)	0.016	0.89 (0.81 −0.99)	-
Model 3[c]	1.00 (ref)	1.37 (1.06 −1.78)	0.97 (0.73 −1.28)	1.02 (0.77 −1.36)	0.525	1.01 (0.89 −1.14)	1.00 (ref)	1.05 (0.86 −1.29)	0.90 (0.73 −1.12)	0.79 (0.63 −0.99)	0.016	0.89 (0.81 −0.99)	0.110

Adjusted hazard ratios and 95% confidence intervals. Analysis using Cox proportional hazards model.

[a] Model 1 was adjusted for age and study area (11 areas).

[b] Model 2 was adjusted as for model 1 plus body mass index, alcohol drinking, total physical activity, history or medication of hypertension/ diabetes/ dyslipidemia, occupation, green tea consumption and coffee consumption.

[c] Model 3 was adjusted as for model 2 plus energy intake.

eTable 9. Multivariable adjusted hazard ratios and 95% confidence intervals of mortality according to energy-adjusted score for adherence to Healthy Meal stratified by occupation in women

	Agriculture/ Fishery/ Forestry (n=9,993)						Others (n=37,357)						*P* for interaction
	Quartile of energy-adjusted score for adherence to Healthy Meal				*P* for trend	1 point increment	Quartile of energy-adjusted score for adherence to Healthy Meal				*P* for trend	1 point increment	
	Q1 (low) (n=2,710)	Q2 (n=2,506)	Q3 (n=2,411)	Q4 (high) (n=2,366)			Q1 (low) (n=9,127)	Q2 (n=9,332)	Q3 (n=9,427)	Q4 (high) (n=9,471)			
Person years	55,048	51,798	49,312	48,454	-	-	176,035	180,023	182,383	182,170	-	-	-
All-cause mortality													
Number of deaths	652	534	513	499	-	-	1,753	1,607	1,447	1,296	-	-	-
Model 1[a]	1.00 (ref)	0.92 (0.82 −1.04)	0.95 (0.84 −1.06)	0.91 (0.81 −1.02)	0.157	0.98 (0.93 −1.03)	1.00 (ref)	0.98 (0.92 −1.05)	0.89 (0.83 −0.96)	0.86 (0.80 −0.92)	<.0001	0.92 (0.89 −0.95)	-
Model 2[b]	1.00 (ref)	0.94 (0.84 −1.06)	0.97 (0.86 −1.09)	0.93 (0.83 −1.05)	0.345	0.99 (0.94 −1.05)	1.00 (ref)	1.01 (0.95 −1.08)	0.95 (0.88 −1.01)	0.92 (0.85 −0.99)	0.006	0.95 (0.92 −0.99)	-
Model 3[c]	1.00 (ref)	0.94 (0.84 −1.06)	0.97 (0.86 −1.09)	0.93 (0.83 −1.05)	0.332	0.99 (0.94 −1.05)	1.00 (ref)	1.01 (0.95 −1.08)	0.95 (0.88 −1.01)	0.92 (0.85 −0.99)	0.006	0.95 (0.92 −0.99)	0.153
Cancer mortality													
Number of deaths	187	179	152	168	-	-	542	564	509	471	-	-	-
Model 1[a]	1.00 (ref)	1.06 (0.86 −1.30)	0.96 (0.78 −1.19)	1.06 (0.86 −1.31)	0.778	1.06 (0.96 −1.18)	1.00 (ref)	1.08 (0.96 −1.21)	0.97 (0.86 −1.10)	0.95 (0.84 −1.07)	0.189	0.97 (0.91 −1.02)	-
Model 2[b]	1.00 (ref)	1.07 (0.87 −1.31)	0.97 (0.78 −1.20)	1.08 (0.87 −1.33)	0.698	1.07 (0.97 −1.19)	1.00 (ref)	1.10 (0.97 −1.23)	1.00 (0.89 −1.13)	0.97 (0.86 −1.10)	0.407	0.98 (0.93 −1.04)	-
Model 3[c]	1.00 (ref)	1.07 (0.87 −1.31)	0.97 (0.78 −1.20)	1.08 (0.87 −1.34)	0.693	1.07 (0.97 −1.19)	1.00 (ref)	1.10 (0.97 −1.23)	1.00 (0.89 −1.13)	0.97 (0.86 −1.10)	0.408	0.98 (0.93 −1.04)	0.123
Cardiovascular disease mortality													
Number of deaths	187	147	165	144	-	-	516	443	386	374	-	-	-
Model 1[a]	1.00 (ref)	0.89 (0.72 −1.11)	1.05 (0.85 −1.29)	0.90 (0.72 −1.12)	0.667	1.00 (0.91 −1.11)	1.00 (ref)	0.93 (0.82 −1.06)	0.83 (0.73 −0.95)	0.88 (0.77 −1.00)	0.014	0.91 (0.86 −0.97)	-
Model 2[b]	1.00 (ref)	0.92 (0.74 −1.14)	1.09 (0.88 −1.35)	0.94 (0.75 −1.17)	0.936	1.02 (0.92 −1.14)	1.00 (ref)	0.98 (0.86 −1.11)	0.89 (0.78 −1.02)	0.96 (0.84 −1.10)	0.302	0.96 (0.90 −1.02)	-
Model 3[c]	1.00 (ref)	0.92 (0.74 −1.14)	1.09 (0.88 −1.35)	0.93 (0.74 −1.16)	0.887	1.02 (0.92 −1.13)	1.00 (ref)	0.98 (0.86 −1.11)	0.90 (0.78 −1.02)	0.96 (0.84 −1.10)	0.312	0.96 (0.90 −1.02)	0.180
Heart disease mortality													
Number of deaths	95	80	81	87	-	-	264	240	188	195	-	-	-
Model 1[a]	1.00 (ref)	0.96 (0.71 −1.29)	1.01 (0.75 −1.35)	1.08 (0.80 −1.44)	0.595	1.10 (0.96 −1.27)	1.00 (ref)	1.01 (0.85 −1.20)	0.81 (0.67 −0.97)	0.91 (0.76 −1.10)	0.090	0.92 (0.85 −1.01)	-
Model 2[b]	1.00 (ref)	1.01 (0.74 −1.36)	1.07 (0.79 −1.45)	1.14 (0.85 −1.54)	0.351	1.14 (0.99 −1.32)	1.00 (ref)	1.07 (0.90 −1.27)	0.88 (0.73 −1.07)	1.02 (0.84 −1.23)	0.645	0.98 (0.90 −1.07)	-
Model 3[c]	1.00 (ref)	1.01 (0.74 −1.36)	1.06 (0.79 −1.44)	1.13 (0.84 −1.53)	0.384	1.14 (0.99 −1.31)	1.00 (ref)	1.07 (0.90 −1.28)	0.88 (0.73 −1.07)	1.02 (0.85 −1.24)	0.680	0.98 (0.90 −1.08)	0.074
Cerebrovascular disease mortality													
Number of deaths	76	57	71	48	-	-	204	167	161	131	-	-	-
Model 1[a]	1.00 (ref)	0.85 (0.60 −1.20)	1.13 (0.81 −1.56)	0.75 (0.52 −1.08)	0.371	0.92 (0.78 −1.08)	1.00 (ref)	0.87 (0.71 −1.06)	0.86 (0.70 −1.05)	0.76 (0.61 −0.95)	0.016	0.86 (0.78 −0.95)	-
Model 2[b]	1.00 (ref)	0.86 (0.61 −1.21)	1.13 (0.82 −1.57)	0.75 (0.52 −1.09)	0.386	0.92 (0.78 −1.08)	1.00 (ref)	0.90 (0.73 −1.11)	0.91 (0.74 −1.12)	0.82 (0.65 −1.02)	0.102	0.90 (0.81 −0.99)	-
Model 3[c]	1.00 (ref)	0.86 (0.61 −1.21)	1.13 (0.81 −1.57)	0.75 (0.52 −1.09)	0.376	0.92 (0.77 −1.08)	1.00 (ref)	0.90 (0.73 −1.11)	0.91 (0.74 −1.12)	0.82 (0.65 −1.02)	0.098	0.90 (0.81 −0.99)	0.542
Respiratory disease mortality													
Number of deaths	65	49	39	34	-	-	123	114	97	65	-	-	-
Model 1[a]	1.00 (ref)	0.87 (0.60 −1.26)	0.71 (0.48 −1.06)	0.62 (0.41 −0.94)	0.014	0.80 (0.66 −0.96)	1.00 (ref)	1.03 (0.80 −1.34)	0.89 (0.68 −1.16)	0.67 (0.49 −0.90)	0.007	0.84 (0.74 −0.96)	-
Model 2[b]	1.00 (ref)	0.89 (0.61 −1.30)	0.76 (0.51 −1.14)	0.67 (0.44 −1.03)	0.047	0.84 (0.70 −1.02)	1.00 (ref)	1.09 (0.84 −1.41)	0.97 (0.74 −1.28)	0.74 (0.54 −1.00)	0.059	0.89 (0.78 −1.02)	-
Model 3[c]	1.00 (ref)	0.89 (0.61 −1.30)	0.76 (0.51 −1.15)	0.68 (0.44 −1.04)	0.053	0.85 (0.70 −1.03)	1.00 (ref)	1.09 (0.84 −1.41)	0.97 (0.74 −1.28)	0.74 (0.54 −1.00)	0.060	0.89 (0.78 −1.02)	0.516

Adjusted hazard ratios and 95% confidence intervals. Analysis using Cox proportional hazards model.

[a] Model 1 was adjusted for age and study area (11 areas).

[b] Model 2 was adjusted as for model 1 plus body mass index, alcohol drinking, total physical activity, history or medication of hypertension/ diabetes/ dyslipidemia, occupation, green tea consumption and coffee consumption.

[c] Model 3 was adjusted as for model 2 plus energy intake.
